# Natural Variation in Preparation for Nutrient Depletion Reveals a Cost–Benefit Tradeoff

**DOI:** 10.1371/journal.pbio.1002041

**Published:** 2015-01-27

**Authors:** Jue Wang, Esha Atolia, Bo Hua, Yonatan Savir, Renan Escalante-Chong, Michael Springer

**Affiliations:** 1 Department of Systems Biology, Harvard Medical School, Boston, Massachusetts, United States of America; 2 Systems Biology Graduate Program, Harvard University, Cambridge, Massachusetts, United States of America; New York University, UNITED STATES

## Abstract

Maximizing growth and survival in the face of a complex, time-varying environment is a common problem for single-celled organisms in the wild. When offered two different sugars as carbon sources, microorganisms first consume the preferred sugar, then undergo a transient growth delay, the “diauxic lag,” while inducing genes to metabolize the less preferred sugar. This delay is commonly assumed to be an inevitable consequence of selection to maximize use of the preferred sugar. Contrary to this view, we found that many natural isolates of *Saccharomyces cerevisiae* display short or nonexistent diauxic lags when grown in mixtures of glucose (preferred) and galactose. These strains induce galactose utilization (GAL) genes hours before glucose exhaustion, thereby “preparing” for the transition from glucose to galactose metabolism. The extent of preparation varies across strains, and seems to be determined by the steady-state response of GAL genes to mixtures of glucose and galactose rather than by induction kinetics. Although early GAL gene induction gives strains a competitive advantage once glucose runs out, it comes at a cost while glucose is still present. Costs and benefits correlate with the degree of preparation: strains with higher expression of GAL genes prior to glucose exhaustion experience a larger upfront growth cost but also a shorter diauxic lag. Our results show that classical diauxic growth is only one extreme on a continuum of growth strategies constrained by a cost–benefit tradeoff. This type of continuum is likely to be common in nature, as similar tradeoffs can arise whenever cells evolve to use mixtures of nutrients.

## Abstract 2

Yeast can anticipate the depletion of a preferred nutrient by preemptively activating genes for alternative nutrients; the degree of this preparation varies across natural strains and is subject to a fitness tradeoff.

## Author Summary

When microorganisms encounter multiple sugars, they often consume a preferred sugar (such as glucose) before consuming alternative sugars (such as galactose). In experiments on laboratory strains of yeast, cells typically stop growing when the preferred sugar runs out, and start growing again only after taking time to turn on genes for alternative sugar utilization. This pause in growth, the “diauxic lag,” is a classic example of the ability of cells to make decisions based on environmental signals. Here we find, however, that when different natural yeast strains are grown in a mix of glucose and galactose, some strains do not exhibit a diauxic lag, or have a very short one. These “short lag” strains are able to turn on galactose utilization—or GAL—genes up to four hours before the glucose runs out, in effect preparing for the transition to galactose consumption. Although such preparation helps strains avoid the diauxic lag, it causes them to grow slower before glucose runs out, presumably because of the metabolic burden of expressing GAL genes. These observations suggest that microbes in nature may commonly face a tradeoff between growing efficiently on their preferred nutrient and being ready to consume alternative nutrients should the preferred nutrient run out.

## Introduction

Natural environments contain complex, time-varying mixtures of nutrients and stresses. Understanding how cells use external cues to maximize growth and survival is key to understanding the evolution and function of regulatory circuits. Gene regulation allows cells to express pathways for specific tasks only in conditions when they are needed, to maximize the benefit of these pathways while minimizing their metabolic cost [1–4]. Regulatory circuits have evolved elaborate behaviors such as bet-hedging, signal integration, and environmental anticipation in response to the complexity of natural environments [5].

A classic example of gene regulation occurs during microbial growth on mixtures of carbon sources. For example, when budding yeast or *Escherichia coli* grow in the sugars glucose and galactose, they first consume glucose, while dedicated signaling mechanisms repress galactose utilization (GAL) genes [6–11]. When glucose has been exhausted, cells temporarily stop growing, induce GAL genes, and start growing again. The transient pause in growth, called the diauxic lag, can last up to several hours.

The diauxic lag is commonly thought to be a consequence of selection to minimize expression of superfluous metabolic pathways when a nutrient that can be more efficiently utilized is available [12–14]. This idea is supported by the observation that cells growing in two sugars that support similar growth rates do not exhibit a diauxic lag [8]. However, recent studies have shown that even in the same nutrient mixture, the length of diauxic lag can vary among experimentally evolved bacterial strains [15, 16] or yeast isolates [17]. In both cases, evolved strains lacking a diauxic shift possessed weaker catabolite repression of secondary carbon pathways than the ancestor. This leads to a fitness cost during growth in the preferred nutrient, but a fitness advantage when the environment shifts rapidly between preferred and alternative nutrients. These results raise the question of whether similar mechanisms and fitness tradeoffs underlie the diauxic lag variation seen in natural yeast isolates [17].

To address this question, we monitored culture density and gene expression in ecologically diverse *Saccharomyces cerevisiae* natural isolates growing in mixtures of glucose and galactose. As expected, we found a spectrum of diauxic lag phenotypes, from strains with nonexistent lags to those with more classical lag times of many hours. Strikingly, the variation in lag time is not due to differences in how fast strains can execute induction of GAL genes, but rather the timing of when they begin to induce. Short-lag strains induce GAL genes up to 4 h before glucose is exhausted, in effect “preparing” for the transition to galactose metabolism. The degree of preparation correlates with the strength of glucose repression; strains that induce GAL genes at higher glucose levels also induce them earlier during diauxic growth. These results suggest that natural variation in catabolite repression is a key determinant of microbial fitness not only during sudden nutrient shifts [17], but also during gradually changing nutrient conditions. Finally, we show that the observed phenotypic variation follows a tradeoff: early GAL gene induction benefits cells by preparing them for glucose exhaustion, but the cost of expressing GAL genes reduces the growth rate while glucose is still present. This tradeoff is likely a general constraint on microbial growth strategies in mixed-nutrient environments.

## Results

### Natural Yeast Strains Vary in Length of Diauxic Lag

**Figure 1:**
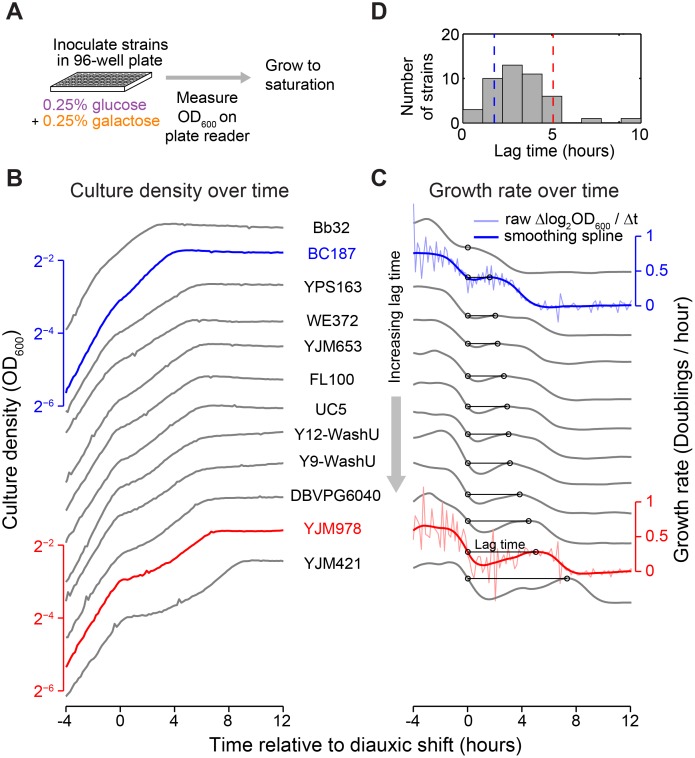
Natural yeast strains vary in length of diauxic lag. (A) Schematic of growth curve experiment in “diauxic growth conditions,” defined as batch culture in synthetic minimal medium with 0.25% glucose and 0.25% galactose. (B) Growth curves (OD_600_ versus time relative to diauxic shift) plotted top-to-bottom in order of increasing diauxic lag. A single replicate growth curve is shown for each of 11 strains with similar growth rates in galactose-only medium. (Growth curves for both replicates of all 43 strains assayed are shown in S1 Fig.) Strains BC187 and YJM978 are highlighted in blue and red, respectively. (C) Smoothed growth rate versus time relative to diauxic shift for the same strains as in (B). Example plots of raw OD_600_ differentials (light blue and light red lines) used to obtain the smoothed growth rate are shown for BC187 and YJM978. Diauxic lag time metric is denoted by horizontal black line with circles (see also S2 Fig.). (D) Histogram of diauxic lag time across all natural isolates assayed. Data used for histogram are the mean of two replicates (Materials and Methods).

We grew 43 *S. cerevisiae* strains in a carbon-limited medium containing 0.25% glucose and 0.25% galactose, the preferred and alternative carbon source, respectively (Fig. 1A). The *S. cerevisiae* strains come from a range of geographical locations and environments [18, 19] and are all prototrophic, allowing us to omit amino acids from the medium and avoid potential complications from their role as alternative carbon sources [20]. Bulk growth of the cells was measured by recording the optical density of each culture every 10 min for 44 h using an automated plate reader (Materials and Methods).

The growth curves generally display an initial phase of fast growth followed by a second phase of relatively slower growth, as expected in a two-sugar mixture (Figs. 1B, 1C, and S1). However, the strains varied in the extent of growth lag, or local minimum in growth rate, between the two growth phases (top versus bottom strains in Fig. 1B and 1C). Some strains (e.g., YJM978) had a long diauxic lag during which the growth rate almost reached zero, whereas some strains (e.g., BC187) had a brief lag period during which even the minimum growth rate was relatively high. Strain SLYG78, a derivative of the commonly used laboratory strain S288C, exhibited a prominent lag phase (S1 Fig.), consistent with previous studies and the traditional understanding of *S. cerevisiae* as having a diauxic growth phenotype [6, 17]

To quantify the variation in diauxic lag, we defined a “diauxic lag time” metric as the time required to reach a strain’s smoothed maximal growth rate in galactose after having dropped below this growth rate during glucose depletion (diauxic lag time shown by horizontal black lines in Figs. 1C and S2B; Materials and Methods). In growth curves that do not have a local growth rate minimum, we defined the lag time as zero (Figs. 1C and S2B). This lag time metric was robust to small differences in culture behavior (*R* ^2^ = 0.96; S2C Fig.) and to the method of calculation (S2D Fig.; Materials and Methods).

We found that diauxic lag time varied continuously in our strains from 0 to 9 h, with a mean of 3.2 h and a standard deviation of 1.6 h (Fig. 1D). The continuous nature of the observed variation was robust to the choice of metric, as a related but distinct growth-curve feature, the minimum mid-diauxic growth rate, also varied continuously and correlated strongly with lag time (*R* ^2^ = 0.71; S2C Fig.). Lag time was not correlated with growth rate in pure glucose or galactose, and even among a subset of strains with similarly high growth rates in galactose-only medium (subset shown in Fig. 1B and 1C), we saw wide variability in the diauxic lag time (S3 Fig.). This suggests that the observed variation is due to differences in metabolic regulation rather than differences in maximal sugar utilization rates.

Several strains displayed no measurable diauxic lag and seemed to transition instantly from glucose consumption to galactose consumption. This implies that these strains can induce GAL genes quickly upon glucose exhaustion, induce GAL genes before glucose exhaustion, or both. To examine these possibilities, we characterized strains YJM978 and BC187, which represent long-lag and short-lag phenotypes, respectively (red and blue curves in Fig. 1).

### Strain BC187 Induces Galactose-Responsive Genes before Glucose Is Exhausted

**Figure 2:**
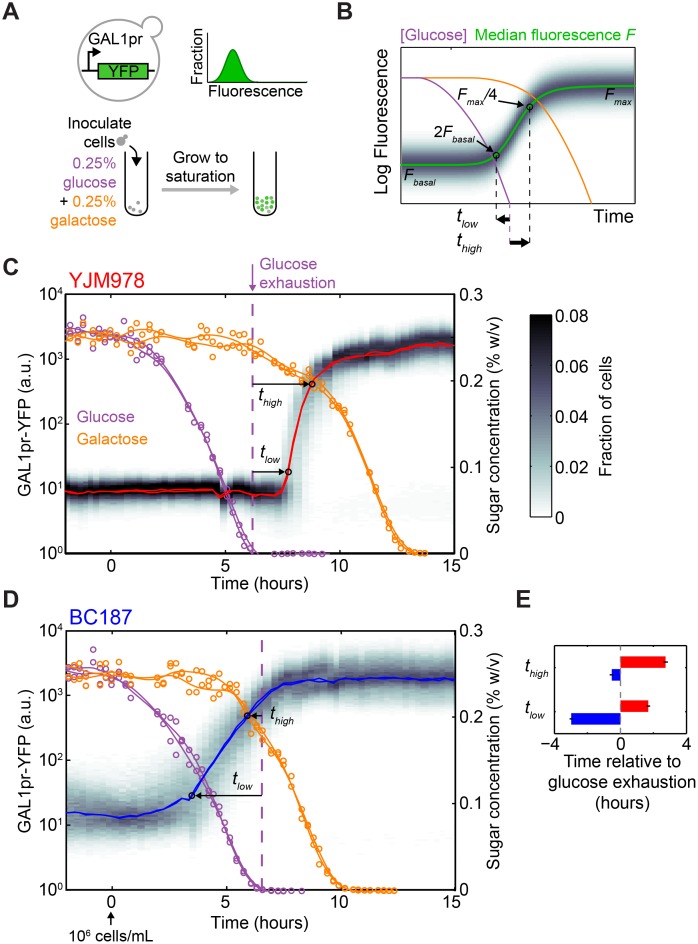
A short-lag strain induces GAL genes hours before the diauxic shift. (A) Top: Schematic of GAL1pr-YFP transcriptional reporter and cartoon of fluorescence distribution as measured by flow cytometry. Bottom: Schematic of diauxic growth GAL gene induction experiment. (B) Definitions of induction metrics, *t*_low_ and *t*_high_, when reporter expression is at low but above-basal or near-maximal levels, respectively. Diauxic growth for strains (C) YJM978 and (D) BC187, with GAL reporter expression distributions (gray shading), GAL reporter median (red line), glucose concentration (purple circles), and galactose concentration (orange circles). Time is defined relative to the moment when the culture achieves a density of 10^6^ cells/ml (S4 Fig.). Purple and orange lines are smoothing-spline fits to glucose and galactose measurements. Dotted purple line indicates time of glucose exhaustion, calculated using a local linear fit (Materials and Methods). Data shown in (B) and (C) represent two replicate experiments. GAL reporter expression distribution is shown for only one of the two replicates. (E) Comparison of induction start time, *t*_low_, and near-maximal induction time, *t*_high_, for YJM978 (red bars) and BC187 (blue bars) cultures. Bars and error bars represent the mean and range, respectively, of two replicates.

We cultured BC187 and YJM978 in 0.25% glucose + 0.25% galactose and monitored GAL pathway expression and glucose and galactose concentrations until saturation, when both sugars were depleted (Fig. 2A; Materials and Methods). We refer to this as a “diauxic growth experiment.” To enable single-cell measurement of GAL gene induction, we integrated a cassette containing yellow fluorescent protein driven by the GAL1 promoter (GAL1pr-YFP)—which has been shown to be a faithful proxy for GAL pathway expression [21–23]—at a neutral chromosomal locus (Fig. 2A, top; Materials and Methods). We measured GAL1pr-YFP expression and extracellular sugar concentration by flow cytometry and enzymatic assay, respectively, over the entire diauxic growth cycle (Fig. 2A, bottom). To quantify the timing of GAL gene induction, we defined *t*_low_ and *t*_high_, respectively, as the time when GAL1pr-YFP expression reached 2-fold above basal levels and 1/4 of maximal levels, relative to the moment of glucose exhaustion (Fig. 2B).

Strain YJM978, which has a long diauxic lag, did not induce galactose-responsive genes until after glucose was exhausted, consistent with the classical understanding of diauxic growth (Fig. 2C; *t*_low_ = 1.7 ± 0.1 h, *t*_high_ = 2.7 ± 0.1 h). In contrast, BC187, which has a short diauxic lag, began GAL reporter induction significantly before glucose exhaustion (Fig. 2D; *t*_low_ = −3.0 ± 0.1 h, *p* = 0.02 by *t*-test on *n* = 2 replicates). Even using the more conservative *t*_high_ metric, BC187 reached near-maximal induction before glucose exhaustion (*t*_high_ = −0.5 ± 0.1 h). Pre-induction of GAL genes by BC187 led to significant galactose consumption, even before glucose was fully exhausted (S5 Fig.). Both strains used glucose and galactose to completion and reached a similar yield (S1 Fig.), indicating that differences in induction time are not due to drastic differences in carbon utilization efficiency. Both strains had undetectable GAL1pr-YFP expression in glucose-only medium (S6 Fig.), ruling out the possibility that galactose metabolism is constitutively active in BC187. In effect, BC187 “prepares” for the diauxic shift by inducing GAL genes before glucose exhaustion.

#### Preparation is a continuous trait among natural yeast isolates

**Figure 3:**
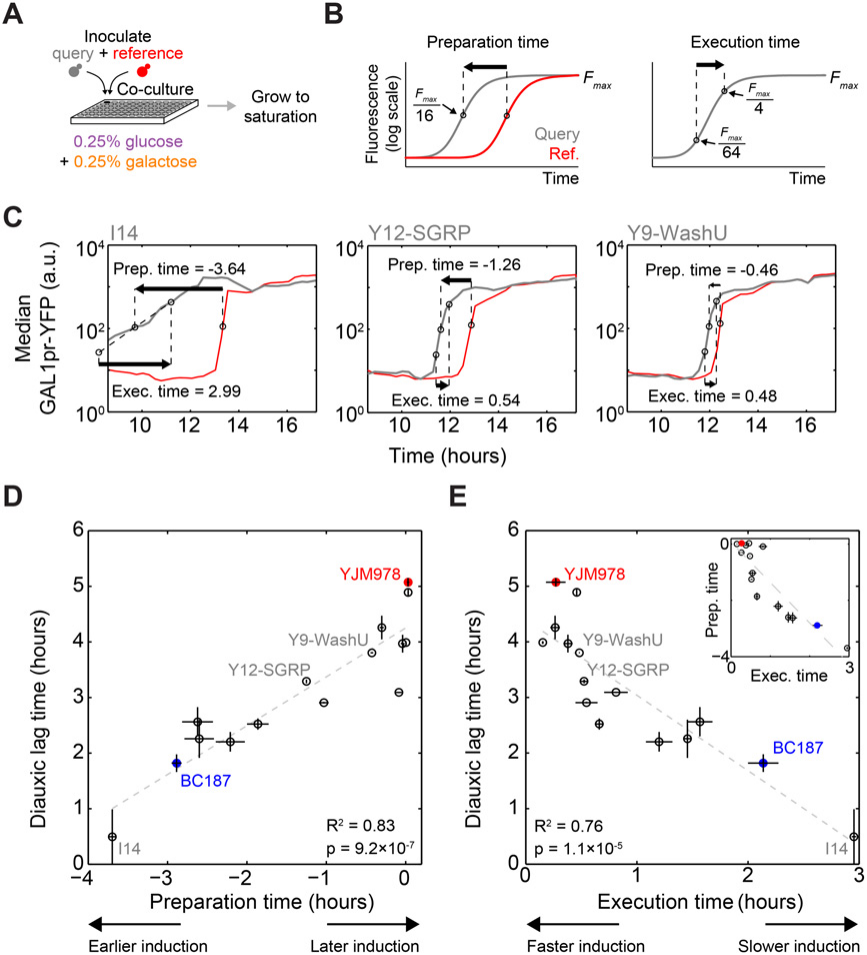
Diauxic lag time is correlated with the start time of GAL gene induction. (A) Schematic of co-culture GAL gene induction experiment. Each of 15 query strains (gray) are co-cultured with reference strain YJM978 expressing constitutive mCherry marker (red), and sampled for flow cytometry every 15 min from mid-exponential phase until saturation. (B) Illustration of how preparation time and execution time metrics are defined. (C) Median GAL1pr-YFP expression versus time for query (gray) and reference (red) strain in three co-cultures selected to illustrate a range of preparation times. Strain I14 had above-basal reporter expression at the start of sampling, so its execution time was computed by linear extrapolation. (D) Scatterplot of diauxic lag time (from Fig. 1) versus preparation time. (E) Scatterplot of diauxic lag time versus execution time. Inset: Scatterplot of preparation time versus execution time. Dotted gray lines in (D) and (E) indicate least-squares linear fits used to calculate coefficients of determination (*R* ^2^) and *p*-values. Data for diauxic lag time are the mean and range of two replicates, and for preparation time and execution time are the mean and SEM of three replicates.

To determine whether GAL gene induction prior to glucose exhaustion is a typical behavior of natural isolates, we integrated a GAL1pr-YFP reporter into 13 additional strains (for a total of 15 strains; see S1 Table) and monitored their expression during diauxic growth (same conditions as in Fig. 2; Materials and Methods). Directly measuring sugar concentrations is laborious and less precise than measuring YFP fluorescence by flow cytometry, so we used YJM978 as a “reference” strain to signal the exhaustion of glucose, and co-cultured it with a “query” strain whose GAL reporter induction kinetics we wanted to assay (Fig. 3A). The reference strain was modified to express a fluorescent marker to distinguish it from the query strain (S7A Fig.; Materials and Methods). To quantify differences in GAL reporter induction time, we defined the “preparation time” as the difference in time between when the query and reference strains reached 1/16 of their maximal median GAL1pr-YFP expression (Fig. 3B and 3C). Preparation time ranged from −3.8 to 0.04 h relative to YJM978, with a mean of −1.3 h, indicating that most strains induce GAL genes earlier than YJM978. The preparation time measured by this method is highly reproducible and robust to the query-to-reference-strain mixing ratio (S7B, S7C, S7E, and S7F Figs.; Materials and Methods). If the degree of preparation determines the extent to which a strain has a diauxic shift, then strains that begin inducing GAL genes earlier should also have a shorter diauxic lag. We found a strong correlation (*R* ^2^ = 0.83, *p* = 9.2 × 10^−7^) between preparation time and the diauxic lag time (Fig. 3D). However, earlier-inducing strains appeared to take longer to reach full induction, or “executed” induction more slowly. We defined the “execution time” as the time required for median GAL1pr-YFP expression to increase from 1/64 to 1/4 of its maximal level (Fig. 3B and 3C). The execution time anticorrelated with preparation time (Fig. 3E, inset) and lag time (Fig. 3E), contradicting the naive expectation that a strain with a shorter diauxic lag would induce GAL genes more quickly. Taken together, our data show that the length of diauxic lag correlates to *when* strains begin to transition to galactose metabolism, not *how fast* they can execute the transition once they begin.

Related studies have observed population heterogeneity of growth rates and gene expression during sudden medium shifts and diauxic growth [17, 23, 24]. In our experimental conditions, strains BC187 and YJM978 did not display bimodality in GAL1pr-YFP expression during diauxic growth (Fig. 2C and 2D). A small number of strains did display bimodal expression at steady state in glucose + galactose (S6 Fig.) and possibly also during diauxic growth (S7D and S7G–S7I Figs.). However, the time window of any bimodality is short compared to the induction time differences between strains (S7G—S7I Fig.; Materials and Methods). Therefore, although single-cell variation is likely an important dimension of regulatory behavior in some strains [17, 23, 24], our analysis of population-level dynamics already captures a major regulatory mode of diauxic growth.

### GAL Gene Induction Kinetics after a Sudden Medium Shift Are Poorly Correlated with Diauxic Lag Time

Our observations above rule out a model of the diauxic lag in which all strains begin inducing upon glucose exhaustion and vary only in how quickly they can reach maximal induction. However, it is possible that instead of inducing at glucose exhaustion, all strains induce when glucose is depleted below a certain threshold and vary in the delay before displaying observable GAL1pr-YFP expression. In this scenario, strains with a short delay between the start of induction and observable GAL1pr-YFP expression would appear to be preparing, whereas strains with a long delay would appear to be inducing only after glucose exhaustion.

**Figure 4:**
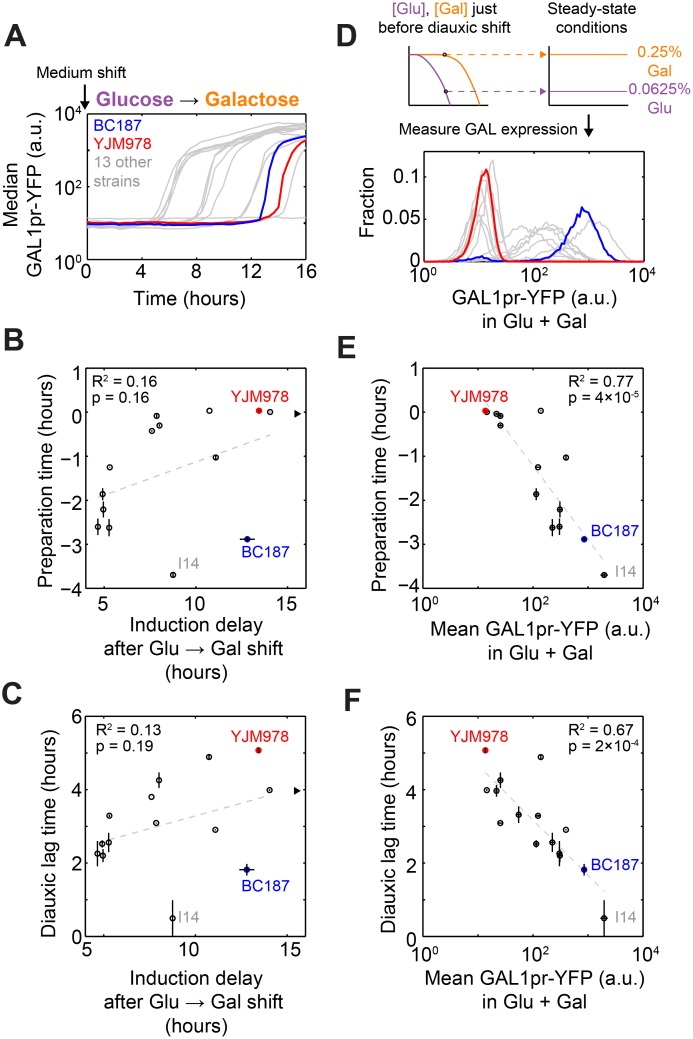
Diauxic lag time correlates poorly with GAL gene induction kinetics but strongly with steady-state GAL gene expression in a glucose–galactose mixture. (A) Median GAL1pr-YFP expression versus time for BC187 (blue line), YJM978 (red line), and 13 other strains (gray lines) after transfer from 2% glucose into 2% galactose. (B) Scatterplot of preparation time (from Fig. 3) versus induction delay after glucose-to-galactose shift, defined as the time until median GAL gene expression reaches 2-fold above basal expression. Black triangle indicates strain YJM981, which did not induce above background during the entire 18-h experiment; this strain was omitted from the *R* ^2^ calculation. (C) Scatterplot of diauxic lag time (from Fig. 1) versus induction delay after glucose-to-galactose shift. (D) Top: Schematic of how sugar concentrations for steady-state measurements were chosen from the diauxic growth experiment. Bottom: Measured steady-state GAL1pr-YFP expression distributions for BC187, YJM978, and 13 other strains in 0.0625% glucose + 0.25% galactose. (E) Scatterplot of preparation time versus mean steady-state GAL1pr-YFP expression from (D). (F) Scatterplot of diauxic lag time versus mean steady-state GAL1pr-YFP expression from (D). Dotted gray lines in (B), (C), (E), and (F) indicate least-squares linear fits used to calculate coefficients of determination (*R* ^2^) and *p*-values. Diauxic lag time data are the mean and SEM of two replicates; preparation time and steady-state GAL1pr-YFP expression are the mean and SEM of three replicates. Induction delay after medium shift is plotted as one replicate.

When cells are grown in glucose, the GAL pathway is repressed [7, 25]. To ask whether differences in glucose de-repression kinetics could explain diauxic lag variation in our natural isolates, we grew strains in 2% glucose and transferred them into 2% galactose. We found significant variation in induction delay, defined as the time until median GAL1pr-YFP expression has increased 2-fold after transfer into galactose (Fig. 4A). Some strains began to induce 5 h after the medium switch, while one strain did not induce even after 18 h. In contrast, the execution time of induction varied only between 0.6 and 1.6 h (S2 Table), suggesting that once glucose repression is relieved, GAL gene expression quickly induces from basal to maximal in all strains. Strikingly, induction delay after glucose-to-galactose shift was a poor predictor of both preparation time (Fig. 4B; *R* ^2^ = 0.16) and diauxic lag time (Fig. 4C; *R* ^2^ = 0.13). In particular, strains BC187 and I14 had short diauxic lags and early preparation times but very long induction delays after a glucose-to-galactose medium shift. When these two strains were omitted from the data, a weak correlation between induction delay after medium shift and diauxic lag time emerged (*R* ^2^ = 0.56; *p* = 0.005), suggesting that glucose de-repression kinetics may play a role in the diauxic lag in our strains, but potentially convolved with a second response such as cell stress.

### Differences in Steady-State Sugar Sensing Explain Variation in Preparation and Diauxic Lag Time

Given that some strains can induce GAL genes in the presence of glucose, we hypothesized that differences in steady-state GAL gene expression in glucose + galactose may underlie differences in preparation. We measured the GAL reporter expression of our natural isolates in 0.0625% glucose + 0.25% galactose to simulate the conditions of a diauxic batch culture just before glucose exhaustion (Fig. 4D). To ensure that we observed the steady-state response of the GAL reporter, we measured induction after cultures reached steady state but before appreciable glucose had been depleted (S8 Fig.; Materials and Methods). We found that steady-state GAL reporter expression in glucose + galactose varied from uninduced to almost maximal (Figs. 4D and S6). On the other hand, all strains were uninduced in glucose-only medium and maximally induced in galactose-only medium (S6 Fig.), suggesting that strains vary not in overall glucose repressibility or inducibility of GAL genes, but in how they integrate signals from both sugars in the mixed environment prior to diauxic shift.

We found that steady-state GAL reporter expression in the glucose–galactose mixture correlated significantly with preparation time (Fig. 4E; *R* ^2^ = 0.77, *p* = 4 × 10^−5^) and diauxic lag time (Fig. 4F; *R* ^2^ = 0.67, *p* = 2 × 10^−4^). In other words, the strains that induced earlier during diauxic growth were those with higher steady-state GAL1pr-YFP expression in glucose + galactose. This suggests that short-lag strains do not suddenly switch GAL genes from “off” to “on” during diauxic growth, but instead express them at quasi-steady-state levels appropriate to the degree of glucose depletion. Consistent with this, the steady-state GAL1pr-YFP expression of these strains is proportional to their expression 3 h before reference strain induction during diauxic growth (S9A Fig.). Furthermore, BC187 grown in three sugar mixtures representing different moments during diauxic growth (0.25% galactose + 0.25%, 0.125%, or 0.0625% glucose) displayed intermediate GAL1pr-YFP expression even after reaching steady state, rather than displaying basal or maximal expression, as would be expected for a switch-like response (S8 and S9B Figs.). Taken together, our data strongly suggest that differences in preparation, and therefore diauxic lag time, are due to differences in the steady-state response of GAL genes to glucose–galactose mixtures.

### All Strains Prepare for Glucose Exhaustion during Diauxic Growth

Comparing the timing of GAL gene induction between diauxic growth and sudden medium shift conditions offers a more sensitive measure of preparation for glucose exhaustion than the diauxic growth experiment alone. Even a long-lag strain like YJM978, which does not show observable GAL1pr-YFP expression until after glucose is exhausted (Figs. 2 and 3), displayed a much shorter induction delay during diauxic growth (*t*_low_ = 1.7 ± 0.1 h; Fig. 2C) than after medium shift from glucose to galactose (induction delay = 12.2 h; Fig. 4A). To directly test whether YJM978 could prepare for glucose exhaustion, we grew it in 0.125% glucose with or without 0.25% galactose and suddenly transferred the cells to galactose. We found that pre-growth in medium containing both galactose and glucose led to an induction delay approximately 1 h shorter than pre-growth in glucose alone, even though GAL1pr-YFP expression was indistinguishable from basal levels in both pre-growth media (S10 Fig.). As YJM978 has one of the longest diauxic lags in our set of strains, these data indicate that all strains prepare for glucose exhaustion to some degree.

### Preparation for Glucose Exhaustion Has an Immediate Cost but Delayed Benefit

The fact that all of our strains prepared for glucose exhaustion by pre-inducing GAL genes suggests that preparation provides a fitness benefit. Consistent with this, strains with shorter diauxic lag times took less time after the diauxic shift to reach saturation (Figs. 1B, 1C, S11A, and S11B). But if preparation is always advantageous, then why don’t all strains display this phenotype? In the diauxic growth experiment of Fig. 2, we noted that the YJM978 culture exhausted glucose 23 ± 4 min before BC187 did (S11C Fig.), even though BC187 eventually exhausted both sugars first. Since BC187 and YJM978 grow at similar rates in glucose-only medium (S3 Fig.), this suggests that BC187 is paying a cost for expressing GAL genes before glucose is exhausted.

**Figure 5:**
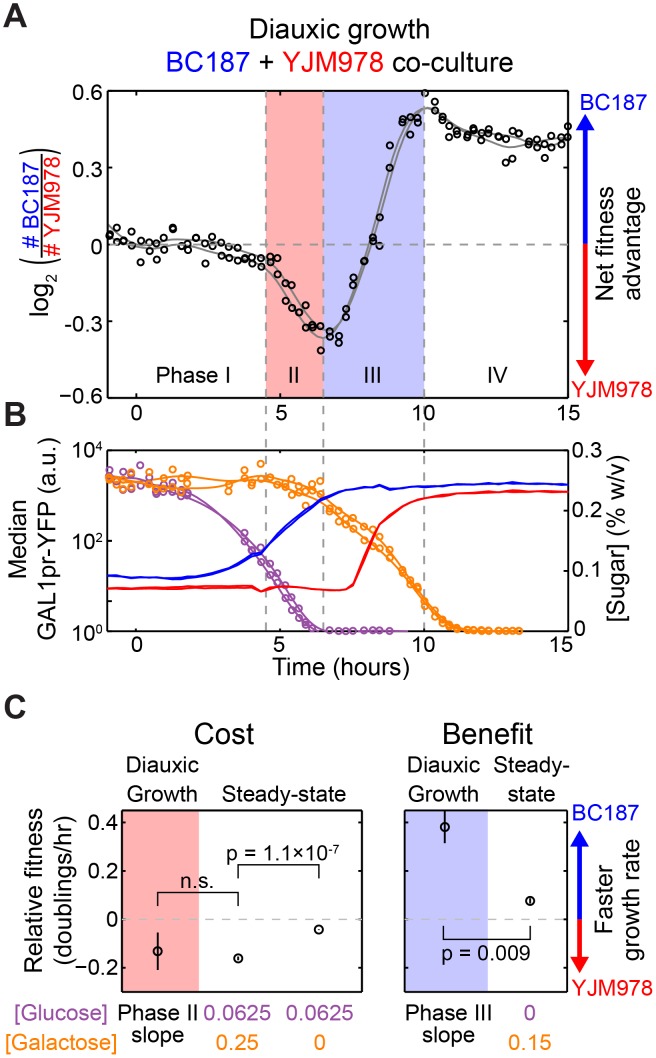
Preparation for glucose exhaustion has upfront cost and delayed benefit. (A) Log2-ratio of BC187 cell number versus YJM978 cell number versus time during diauxic growth in two replicate co-cultures. A positive value on the vertical axis at any given moment indicates that BC187 has divided more times than YJM978 since time = 0, and therefore has a net fitness advantage. Raw data (black circles) and smoothing splines (gray curves) are shown for two replicates. (B) Median GAL1pr-YFP expression of BC187 (blue lines) and YJM978 (red lines), glucose concentration (purple circles and lines), and galactose concentration (orange circles and lines) from (A). Vertical dotted gray lines in (A) and (B) demarcate four phases of relative fitness and GAL1pr-YFP expression during the experiment (see Results). (C) Comparison of growth rate differences during diauxic growth versus steady-state sugar conditions. Data points on shaded backgrounds and labeled “diauxic growth” represent the slope of the data in (A) during Phase II (pink background) and Phase III (blue background). Positive values indicate that BC187 grows faster than YJM978. Data are the mean and SEM of *n* = 6 (Phase II) or *n* = 14 (Phase III) discrete derivatives in the shaded regions from (A). Points on a white background and labeled “steady-state” are computed from the same data as in S12C Fig., and represent the mean and SEM of 3–6 replicates. *p*-Values are computed by two-sample *t*-test.

To directly measure potential costs and benefits experienced by BC187 during diauxic growth, we performed a competitive fitness assay by co-culturing BC187 and YJM978 under diauxic growth conditions. In addition to GAL1pr-YFP reporter expression, we also monitored the relative abundance of the two strains by tagging them with constitutive fluorophores (Fig. 5A; Materials and Methods). We plotted the log ratio of BC187 to YJM978 cell counts versus time and found four different phases of relative fitness during a diauxic growth cycle (Fig. 5A). Initially, when both sugar concentrations are high, both strains exhibit low GAL1pr-YFP expression (Fig. 5B, Phase I) and grow at comparable rates (growth rate difference less than 0.062 doublings/hour with 95% confidence). When glucose is depleted below 0.1%, BC187 displays increased GAL1pr-YFP expression while YJM978 does not (Fig. 5B, Phase II). During this phase, BC187 has a significant fitness disadvantage of −0.17 doublings/hour relative to YJM978 (Fig. 5A and pink-shaded point in Fig. 5C; *p* = 0.0025 for non-zero slope by *t*-test). After glucose exhaustion, YJM978 begins to induce GAL1pr-YFP (Fig. 5B, Phase III), and here BC187 has a significant fitness advantage of 0.38 doublings/hour relative to YJM978 (Fig. 5A, blue-shaded point in 5C; *p* = 7.7 × 10^−5^ for non-zero slope by *t*-test). Once GAL1pr-YFP is fully induced in both strains, the relative fitness is again comparable (Fig. 5A, Phase IV; fitness difference less than 0.06 doublings/hour with 95% confidence).

This experiment shows that BC187 grows more slowly than YJM978 just before glucose exhaustion (Fig. 5A, Phase II). To rule out that this is due to differences in utilization of low glucose concentrations unrelated to GAL regulation, we measured the absolute growth rates of the two strains in 0.0625% glucose with or without 0.25% galactose, where sugar concentrations were held constant by frequent dilution (S12 Fig.; Materials and Methods). We found that BC187 grew at 0.62 doublings/hour in glucose alone, but significantly slower, at 0.51 doublings/hour, in glucose + galactose (S12C Fig.; *p* = 3.2 × 10^−4^ by *t*-test on *n* = 3–6 replicates per condition). YJM978 had the same growth rate of 0.67 doublings/hour in both conditions. Neither strain showed GAL1pr-YFP expression in glucose alone, but in glucose + galactose, BC187 displayed near-maximal induction while YJM978 remained at background (S12D Fig.). These results correspond to a relative fitness of BC187 to YJM978 of −0.043 doublings/hour in glucose alone and −0.16 doublings/hour in glucose + galactose. Only the latter is comparable to the fitness difference of −0.13 doublings/hour just prior to glucose exhaustion during diauxic growth (Fig. 5C, left panel). Therefore, the fitness difference prior to glucose exhaustion is due to a *steady-state* cost of BC187’s early response to galactose.

In principle, the fitness difference after glucose exhaustion (Fig. 5A, Phase III) could be due to differences in galactose utilization rather than being a benefit from pre-induction of GAL genes. To rule this out, we measured the steady-state relative fitness of the strains in 0.15% galactose (S12C Fig.), corresponding to the carbon conditions just after glucose exhaustion, when BC187 has its largest fitness advantage (0.38 doublings/hour) over YJM978 (Fig. 5A and 5B, Phase III). When galactose was held constant at 0.15%, BC187 had only a 0.076-doublings/hour advantage over YJM978 (Figs. 5C and S12C). This steady-state relative fitness is significantly lower than the fitness difference during Phase III of diauxic growth (*p* = 0.009 by *t*-test; Fig. 5C, right panel), showing that the majority of the fitness benefit after glucose exhaustion during diauxic growth is *kinetic*, not steady state.

These results indicate that GAL pathway expression has a strong influence on growth rate in both constant and time-varying sugar environments. If this is a direct result of GAL gene activity, then cells from the same population with non-genetic variation in GAL gene expression should also exhibit different growth rates. To test this, we performed time-lapse microscopy to measure the growth rate and GAL reporter expression of BC187 cells growing in 0.125% glucose + 0.25% galactose, a partially inducing condition (S13 Fig.; Materials and Methods). To maximize the dynamic range of GAL reporter expression of the observed cells, we pre-induced cells to low, medium, and high GAL1pr-YFP expression by culturing them in 0.125% glucose, 0.125% glucose + 0.25% galactose, and 0.25% galactose, respectively. We found that growth rate and GAL1pr-YFP expression displayed a significant negative correlation across cells of the same population, regardless of the pre-culture medium (S13B Fig.). Furthermore, cell populations pre-induced to higher GAL1pr-YFP levels displayed lower growth rates than populations pre-induced to lower GAL levels (S13C Fig.). Therefore, the fitness differences between bulk cultures of different strains may be due to effects of GAL gene expression at the single-cell level.

### Synthetic Expression of GAL Genes Recapitulates Costs and Benefits

**Figure 6:**
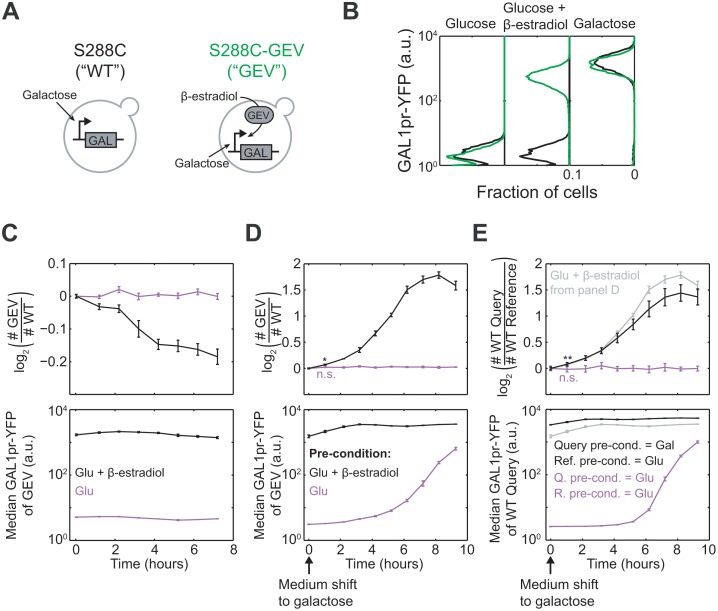
Synthetic induction of GAL genes is costly in glucose but beneficial during transition to galactose. (A) Strains S288C (WT) and S288C-GEV, a congenic strain expressing the GEV protein, were used. Both WT and GEV strains induce GAL genes in response to galactose; strain GEV also induces GAL genes in response to β-estradiol. For technical reasons, two variants of the WT strain were used; each strain is haploid and its HO locus has been replaced with either a GAL1pr-YFP reporter (SLYA39) or a constitutive mCherry segmentation marker (SLYA32). (B) GAL1pr-YFP expression histograms of strains WT (SLYA39) (black) and GEV (green) at steady state in 2% glucose, 2% glucose + 30 nM β-estradiol, or 2% galactose. The same concentrations were used in the following experiments. (C) Top: log_2_ ratio of GEV to WT (SLYA32) cell counts during steady-state co-culture in glucose (purple) or glucose + β-estradiol (black). Bottom: median GAL1pr-YFP expression of strain WT during this experiment. (D) Top: log_2_ ratio of GEV to WT (SLYA32) cell counts upon sudden shift to galactose, after pre-growth in glucose (purple) or glucose + β-estradiol (black). Bottom: median GAL1pr-YFP expression of strain GEV during this experiment. (E) Top: log_2_ ratio of cell counts of WT strain SLYA39 and SLYA32 after pre-growth in different conditions and shift to galactose medium. Before the medium shift, the strains were either both preconditioned in glucose (purple), or the query strain (SLYA39; numerator of log ratio) was preconditioned in galactose while the reference strain (SLYA32; denominator of log ratio) was preconditioned in glucose (black). The black line from (D) is reproduced in gray in (E) to compare synthetic and “natural” pre-induction of GAL genes. Bottom: median GAL1pr-YFP expression of the query strain (SLYA39) during this experiment. Data in (C–E) are mean and SEM of three replicates. *p = 0.008, **p = 0.01 for change in log_2_ strain ratio by two-sample *t*-test. n.s., not significant (*p* > 0.05).

Given the long-established role of GAL genes in performing and regulating galactose metabolism [10], our findings strongly suggest that GAL gene expression causes the observed costs and benefits. Nevertheless, it is possible that unknown genes outside of the GAL pathway also mediate cellular responses to the environments we studied. To show that expression of GAL pathway genes alone is sufficient to produce a fitness cost and a benefit, we introduced the chimeric transcription factor GEV into the S288C lab strain background (S288C-GEV; Fig. 6A) [26, 27]. The presence of β-estradiol, an otherwise inert compound in yeast, triggers the GEV protein to activate genes responsive to the GAL pathway activator GAL4p [28, 29]. Therefore, S288C-GEV cells grown in glucose + β-estradiol will express all the inducible genes in the GAL pathway, as well as a GAL1pr-YFP reporter we integrated into this strain (Fig. 6B; Materials and Methods). As expected, we found that S288C-GEV had a fitness cost relative to an unmodified S288C strain when grown in glucose + β-estradiol (Fig. 6C, top panel, black line). This cost was absent in glucose-only medium (Fig. 6C, top panel, purple line), where S288C did not express GAL genes (Fig. 6C, bottom panel).

We found that S288C-GEV pre-induced in glucose + β-estradiol had an advantage over uninduced S288C when transferred suddenly to galactose medium (Fig. 6D). We saw a similar advantage when strain S288C was “naturally” pre-induced by being grown in galactose, and then mixed with uninduced S288C and shifted together to galactose (Fig. 6E). Therefore, induction of GAL genes recapitulates the benefits of galactose pre-growth in preparing cells for a transition to galactose. Surprisingly, the advantage of pre-induction (Fig. 6D and 6E, slope of black line) is largest 3–6 h after medium shift rather than immediately. However, this delay is seen for both synthetic and “natural” pre-induction, suggesting that it is due to stresses of the medium shift unrelated to sugar metabolism (Materials and Methods). In fact, even the immediate advantage of pre-induction is significant; by 1 h after the shift to galactose, the synthetically pre-induced strain had made 0.068 more doublings than the non-pre-induced strain (*p* = 0.008; asterisk in Fig. 6D). This advantage is almost identical to the immediate advantage conferred by “natural” pre-induction (Fig. 6E, gray and black lines). Therefore, expression of GAL genes alone is sufficient to cause a fitness cost in glucose-containing environments and a fitness benefit during transitions to galactose.

### Tradeoff between Costs and Benefits of Preparation Is a General Constraint

**Figure 7:**
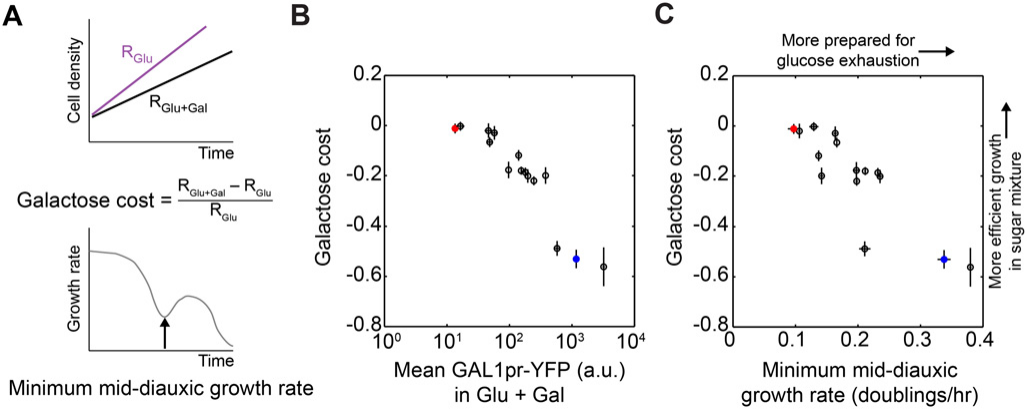
Tradeoff between costs and benefits of preparation underlies natural variation in GAL pathway expression. (A) Illustration of how galactose cost (top) and the minimum mid-diauxic growth rate (bottom) are defined (see also S2 and S14 Figs., and Materials and Methods). Glucose and glucose + galactose conditions indicate 0.03125% glucose and 0.3125% glucose + 0.25% galactose media, respectively. (B) Scatterplot of galactose cost versus mean GAL1pr-YFP expression at steady state in glucose + galactose. Data points are mean and SEM of *n* = 3 replicates. (C) Scatterplot of galactose cost versus minimum mid-diauxic growth rate. The latter is computed from the growth curves shown in Figs. 1 and S1. Data points are the mean and SEM of *n* = 3 replicates (galactose cost) or mean and range of *n* = 2 replicates (minimum rate).

Our data indicate that BC187 pre-induces GAL genes at a cost before the diauxic shift but reaps a benefit afterward, whereas YJM978 minimizes its preparation cost at the expense of experiencing a growth lag. To see whether this tradeoff also constrains our other natural isolates, we assayed 15 strains to determine the cost they incur in responding to galactose while glucose is present. We defined the “galactose cost” of each strain as the relative difference in its steady-state growth rate in glucose + galactose versus glucose only, or specifically, as (*R*_glu+gal_ − *R*_glu_)/*R*_glu_, where *R*_glu+gal_ represents the growth rate in 0.03125% glucose + 0.25% galactose and *R*_glu_ represents the growth rate in 0.03125% glucose. Galactose cost ranged from 0 to −0.6, meaning that a strain may grow up to 60% slower simply because galactose is present in addition to glucose. The cost experienced by a given strain increased with its GAL1pr-YFP expression in glucose + galactose (Fig. 7B), suggesting that the growth rate reduction is due to expression or activity of GAL genes. Additionally, when the cost measurement was repeated in 0.125% glucose + 0.25% galactose, a condition that elicits lower GAL1pr-YFP expression from most strains, the magnitude of galactose cost also decreased (S14 Fig.). These results confirm the presence of a tradeoff: no strain can partially induce GAL genes without also experiencing a decrease in growth rate.

To further illustrate this tradeoff, we used the minimum mid-diauxic growth rate (“minimum rate”) as a direct metric for the benefit of preparation (Fig. 6A, bottom). This metric is correlated with lag time and intuitively captures why preparation is beneficial: the more prepared a strain is, the higher its growth rate will be just after glucose exhaustion (S2C Fig.). Furthermore, the minimum rate is not correlated with the growth rate in glucose or galactose alone, and therefore is not convolved with steady-state metabolic differences (S3 Fig.). As expected, we found a negative correlation between preparation cost and minimum rate (Fig. 7C). Our model strains for short-lag and long-lag phenotypes, BC187 and YJM978, appeared near the extremes of this tradeoff, with the phenotypes of most other strains in between.

## Discussion

### “Why No Lag Phase?”: An Old Problem Revisited Again

A recent study by New et al. found that yeast strains that evolved to respond quickly to sudden glucose-to-maltose (i.e., preferred-to-alternative sugar) transitions tended to also have shorter diauxic lags [17]. These evolved isolates acquired mutations that weakened carbon catabolite repression, so that maltose utilization (MAL) genes were partially induced in otherwise repressing glucose levels. New et al. found that partial MAL gene expression is costly when glucose is available, but enables cells to resume growth more quickly when the environment changes suddenly from glucose to maltose.

Here we confirm the link between diauxic lag duration and glucose repression found by New et al., and observe an analogous expression cost in the GAL pathway, consistent with other reports [14, 30]. Additionally, we extend the previous results in two ways. First, we show that variation in glucose repression leads to a spectrum of GAL pre-induction phenotypes during diauxic growth, and that this “preparation” is mediated by steady-state sugar-sensing rather than by induction or de-repression kinetics. Second, we demonstrate that the same cost–benefit tradeoff that constrains lab evolution in an environment of sudden nutrient shifts also applies to natural isolates in gradually depleting nutrient mixtures. Overall, our results suggest that the mechanisms and selective forces that New et al. found in evolved strains are very likely also relevant in nature.

### Preparation Arises from Weak Catabolite Repression during Gradual Glucose Depletion

Preparation for the diauxic shift can be attributed to two key features of the yeast GAL pathway. First, some strains express GAL genes at relatively high levels in glucose–galactose mixtures [31]. This partial induction has the effect of allowing cells to anticipate sudden nutrient shifts, which New et al. also hypothesized underlay differences in diauxic lag duration [17]. However, it is not obvious a priori whether partial inducibility of GAL genes is physiologically relevant during diauxic growth, because glucose depletion must be slow relative to the timescale of response of GAL genes in order for significant pre-induction to occur. Our experiments demonstrate this second feature, and show that cells are indeed able to initiate (or continue) GAL gene induction during, and not only after, glucose depletion. For example, in our culture conditions, strain BC187 took 4.1 h and YJM978 took 3.3 h to deplete glucose from 0.2% to 0%, while both strains could execute induction from 1/64 to 1/4 of maximal expression in less than 2 h. Even long-lag strains, which do not display observable induction prior to the diauxic shift, still begin to induce sooner during diauxic growth than after a sudden nutrient shift, suggesting that all strains can prepare for glucose depletion. These findings contribute to growing evidence that batch culture is a continuous dynamical process and that this feature plays an important role in cellular regulation [32, 33].

### Induction Timing, Not Speed, Underlies Variation in Diauxic Lag

Previous studies have described differences in diauxic lag in terms of how quickly strains can transition from preferred to non-preferred nutrient metabolism [15–17]. We found that in a gradually depleting glucose–galactose mixture, “fast” or “slow” changes in growth rate were not due to “fast” or “slow” induction of GAL genes from basal to maximal, nor high basal induction, but rather were due to “early” or “late” initiation of induction relative to glucose exhaustion. This clarifies a distinction between induction “speed” and “timing” that has not been addressed explicitly in previous work on diauxic growth.

New et al. observed a correlation between diauxic growth phenotype and growth delay after a glucose-to-maltose shift [17], suggesting that common mechanisms underlie the behavior of cells in sudden and gradual nutrient shifts. We observed that diauxic lag duration was only weakly correlated with induction delay after a sudden glucose-to-galactose shift (Fig. 4A–4C), and instead that diauxic lag was more strongly correlated to preparation time and partial GAL reporter expression (Figs. 3 and 4D–4F). This discrepancy may be due to differences in our experimental systems, and suggests that our strains may experience stress after the glucose-to-galactose shift incurred by sudden loss of a metabolizable carbon source (Materials and Methods).

### Preparation as a Widespread Regulatory Strategy

Other examples of preparation have recently been described in microbes encountering specific sequences of nutrients or stresses. For example, when *E. coli* bacteria encounter either heat shock or low oxygen, they induce both heat-responsive and low-oxygen-responsive genes, presumably an adaptive response when entering the warm, oxygen-deprived mammalian gut [34]. The co-regulation was decoupled by lab evolution under repeated heat shock in constant high oxygen, suggesting that the secondary response was neutral or costly when not needed. Anticipatory responses can also be asymmetric. When domesticated yeast encounter stresses typical of the early stages of fermentation, they acquire resistance to later stresses; however, later stresses do not trigger resistance to early ones [35]. These results demonstrate that simple biochemical circuits can evolve the ability to anticipate environmental changes when the environmental cues occur in a predictable temporal sequence [36]. We now have shown that low or decreasing levels of a preferred nutrient can serve as a predictive cue for eventual depletion. Since this is inevitable when cells deplete a mixture of nutrients at unequal rates, and mixed-nutrient environments are ubiquitous in nature, environmental anticipation may be a more widespread regulatory strategy than previously recognized.

### Natural Variation in Diauxic Lag May Result from a Tradeoff between Costs and Benefits

To be considered a meaningful example of preparation, a response must be beneficial in the future but neutral or costly in the present [35, 36]. We showed that anticipatory GAL gene induction is costly—specifically, that many strains grow faster in glucose-only medium than in medium containing the same concentration of glucose plus an inducing concentration of galactose. The magnitude of the cost is correlated to the degree of GAL reporter expression across genetically diverse natural isolates, as well as across cells of the same strain with non-genetic expression variation. This cost can likely be attributed to the expression or activity of GAL pathway genes, because a strain that synthetically induces GAL genes in an otherwise non-inducing environment also exhibits a growth defect. These results rule out the possibility that strains induce GAL genes in glucose + galactose because it provides additional energy and thus a selective advantage.

The cost of GAL gene induction confirms part of the traditional rationale for the diauxic lag: strains that maintain stringent repression of alternative sugar pathways gain an advantage by maximizing their growth rate on glucose. On the other hand, we show that pre-induction also has a benefit that can sometimes more than compensate for its cost. Simply by being able to grow when glucose runs out, BC187 is able to double its population size over 3 h while YJM978 undergoes a lag phase. This benefit is recapitulated when synthetically pre-induced cells are shifted from glucose to galactose medium. The prevalence of short-lag phenotypes among natural strains shows that diauxic lag is by no means an inevitable phenotype in nature, and may be selectively advantageous only in certain conditions.

We find that strains seem to face a tradeoff between fast growth on glucose and readiness to grow on galactose when glucose runs out. In principle, these goals need not be in conflict, and given the countless ways that genetic variation can tune growth rates and gene expression, perhaps evolution can optimize multiple traits simultaneously. In fact, a naive analysis reveals no tradeoff between our diauxic growth metrics and unnormalized growth rates in glucose or galactose (S3 Fig.), consistent with a similar observation by New et al. [17]. Therefore, although the correlations that we observed across natural isolates suggest that there could be a causal relationship between GAL gene regulation and fitness consequences during diauxic growth, definitive proof of this idea requires future work incorporating genetic and mechanistic analyses.

Given these caveats, it is nevertheless striking that we do observe a tradeoff between minimum diauxic growth rate and a galactose cost metric normalized for baseline growth rate differences in glucose. Like other examples of biological tradeoffs [2, 3, 37], our observation suggests the presence of underlying constraints despite substantial variation in other traits. In our strains, this constraint is likely the combination of an upper “speed limit” on how quickly GAL gene induction can be executed and an unavoidable cost of pre-induction.

### Bet-Hedging, Mixed Strategies, and Optimal Growth

In this study, we focused on the timing of induction of entire cell populations during diauxic growth. Some of our natural isolates displayed bimodal GAL reporter induction, similar to that of lab-evolved isolates, suggesting that the core phenomenon of preparation may be further modulated by heterogeneity across single cells. In fact, a different lab strain, W303, has been found to implement both early and late induction strategies simultaneously in subpopulations of the same culture [23], reminiscent of microbial “bet-hedging” observed in other contexts [38–40]. This “mixed strategy” can be evolutionarily stable, as mutants with unimodal GAL gene induction are unable to invade the bimodal wild-type (WT) strain in glucose–galactose mixtures [41]. Similar population diversification during diauxic growth has been observed in bacteria [24, 42, 43]. Additionally, cellular decisions in nutrient mixtures can be influenced by epigenetic memory [22, 44, 45] and inter-species signaling [46, 47]. An important goal of future investigation will be determining the relative importance of the contributions of these different processes to cellular decision-making in complex natural environments.

## Materials and Methods

### Strains and Media

Natural isolate yeast strains were obtained from multiple sources: 23 strains were part of the Saccharomyces Genome Resequencing Project and were obtained from the National Collection of Yeast Cultures [18]; 18 strains were obtained from the Fay lab at Washington University [19]. Strain Bb32 was obtained from the Broad Institute [48]; strain SLYG78 was constructed for this study. Some strains were obtained in duplicate, which we indicate by affixing “-SGRP” or “-WashU” to the strain name. One of these, Y12, displayed reproducibly different diauxic growth phenotypes depending on the source collection—this may be due to strain mislabeling (S1 Table) [49, 50]. All strains were homozygous diploid and prototrophic.

Growth curves were performed on 43 strains, and a subset of 15 natural isolates was chosen for subsequent analyses. A full strain list, as well as detailed genotypes of the 15-strain subset, can be found in S1 Table. With the exception of SLYG78, the subset strains were transformed with vector SLVA63 or SLVD02 digested with NotI, which replaces the chromosomal HO locus with GAL1pr-YFP linked to the kanMX4 or hphNT1 selection marker, respectively. Deletion of HO does not affect growth rate [51]. Strain SLYG78 was constructed by transforming S288C-lineage haploid strains FY4 and FY5 [52] with GAL1pr-YFP and TDH3pr-mTagBFP2 (vectors SLVD02 and SLVD13), respectively, and mating them to obtain a diploid. Strains BC187 and YJM978 were transformed a second time with SLVA19 or SLVA06, which replaces the second HO locus with TDH3pr-mTagBFP or TDH3pr-mCherry linked to natMX4, respectively. These strains are designated BC187yb and YJM978ym in this section and in the Supporting Information, but simply BC187 and YJM978 in the main text for clarity. Strain BC187ym was used for time-lapse microscopy experiments (S13 Fig.) instead of BC187yb (see “Single-Cell Measurements by Time-Lapse Microscopy”); the two strains are identical other than the fluorescent protein they express. Strains for synthetic GAL gene induction via GEV are described below. All yeast transformations were done by the standard lithium acetate procedure [53].

All experiments were performed in synthetic minimal medium, which contains 1.7 g/l yeast nitrogen base (YNB) (BD Difco) and 5 g/l ammonium sulfate (EMD Millipore), plus carbon sources. YNB contains no amino acids and extremely small amounts of other carbon-containing compounds, and therefore the added sugars comprise the sole carbon source. For diauxic growth experiments (Figs. 1–3), the synthetic minimal medium base was supplemented with 2.5 g/l glucose (EMD Millipore) and 2.5 g/l galactose (Sigma) to obtain 0.25% glucose + 0.25% galactose w/v. We chose a 1:1 mixture of sugars to maximize the amount of growth curve data in both diauxic growth phases, and a total carbon concentration of 0.5% w/v because it is the highest that can be completely exhausted in synthetic minimal medium before non-carbon nutrients become yield-limiting. Unless noted otherwise, cultures were grown in a humidified incubator (Infors Multitron) at 30°C with rotary shaking at 230 rpm (tubes and flasks) or 999 rpm (deep 96-well plates).

### Growth Curves and Diauxic Lag Time Metric

Growth curves (Fig. 1) were obtained using an automated robotic workcell in a room maintained at 30°C and 75% humidity. Strains were cultured in 150 μl of medium in optical-bottom 96-well plates (CELLTREAT). Plates were cycled between a shaker (Liconic) and a plate reader (PerkinElmer EnVision) using a robotic arm (Caliper Life Sciences Twister II), and absorbance at 600 nm (OD_600_) was measured for each plate approximately every 10 min for up to 48 h. In the humidity-controlled room, evaporation of medium was negligible within this time. Strains to be assayed were pinned from glycerol stock onto YPD agar and incubated for 16 h, and then pinned into 600 μl of liquid YPD and incubated another 16 h. These cultures were diluted 1:200 into 600 μl of synthetic minimal medium + 0.5% glucose and grown for 8 h, and finally diluted 1:300 into synthetic minimal medium + 0.25% glucose + 0.25% galactose for growth curve measurements. The final inoculation was performed into two different plates; these replicate growth curves were nearly indistinguishable for all strains (S1 Fig.).

Analysis of growth curve data was performed in MATLAB using custom-written code. Raw OD_600_ readings were background-corrected by subtracting the median OD of 5–10 media-only wells on each plate. OD increased linearly with culture density in the density range of our cultures (S2A Fig.). The OD of a typical saturated culture in our experiment was approximately 0.3, which corresponds to 5 × 10^7^ cells/ml. To analyze the diauxic lag, a smoothed growth rate was obtained by log_2_-transforming the data, computing discrete derivatives between consecutive data points as (OD_*i*_ − OD_*i*−1_)/(*t*_i_ − *t*_i−1_), and fitting the derivatives to a cubic spline using the MATLAB function csaps with a smoothing parameter of 0.75. This smoothed derivative represents the instantaneous growth rate in units of doublings/hour. The diauxic lag time metric was computed as the difference in time between the last local maximum in the smoothed growth rate and the previous point where the culture had the same growth rate; the earlier point was also used as the time of diauxic shift (Figs. 1 and S2B). The minimum mid-diauxic growth rate was computed as the minimum value of the smoothed growth rate between these two times (S2B Fig.). In strains that did not have a local minimum in the smoothed growth rate, we defined the diauxic lag as zero and the minimum mid-diauxic growth rate as the value of the smoothed growth rate at its inflection point between the two growth phases; this inflection point was also used as the time of diauxic shift (S2B Fig., strain Bb32). Similar results were obtained if the two metrics were calculated using a sliding-window average on the discrete derivatives instead of a smoothing spline (S2D Fig.). We chose the smoothing-spline method because it facilitated calculation of a second derivative to allow identification of inflection points in the growth rate (S2B Fig., red lines).

To obtain growth rates in glucose or galactose (S3 Fig.), additional growth curves were performed as above, except the final culture medium contained 0.5% glucose alone or 0.5% galactose alone. The exponential growth rate was extracted from these data as the mean growth rate between when OD_600_ = 2^−6^ and OD_600_ = 2^−4^ (or, equivalently, when culture density was approximately 1/16 and 1/4 of saturation, respectively).

### Flow Cytometry and Sugar Assays on Diauxic Batch Cultures

We assayed the gene expression and sugar consumption of BC187yb, YJM978ym, or a co-culture of the two during diauxic growth (Figs. 2 and 5) by inoculating them from single colonies into liquid YPD, incubating for 16 h, mixing 1:1 by volume if co-culturing, and then diluting 1:100–1:500 into 2% raffinose and growing for 20 h to ~1.5 × 10^6^ cells/ml. The raffinose cultures were pelleted by centrifugation, washed once, and then resuspended in 0.25% glucose + 0.25% galactose medium in two replicate cultures of 50 ml each. The cultures were incubated in flasks at 30°C with shaking, and a sample was removed every 15 min until saturation, about 18 h. Some sample was placed on ice and diluted 1:2–1:100 in Tris-EDTA (pH 8.0) and read immediately on a Stratedigm S1000EX cytometer. The flow cytometer injected a defined volume, so we could estimate the absolute culture density (S4A Fig.). The remaining sample was filter-sterilized using a Pall 0.2-μm filter plate, and the flow-through stored at −20°C. Media flow-throughs were later thawed and assayed for glucose and galactose concentrations by mixing with a sugar-specific oxidase (Megazyme) and measuring the absorbance of the reaction at 340 nm. A standard curve of known sugar concentrations was also assayed and used to infer concentration from absorbance. We expect YFP signal to change 1 h slower than GAL1 protein levels, because of fluorophore maturation time [54]. This may be why galactose decreases slightly in the YJM978 culture before GAL1pr-YFP increases (Fig. 2D). However, since all strains have the same reporter, this should not affect induction time differences between strains.

### Analysis of Flow Cytometry and Sugar Time-Course Data

Flow cytometry data were analyzed using custom MATLAB code. In co-culture experiments, a 2-D Gaussian mixture model (using the gmdistribution class) was fit to mCherry and side-scatter signal to segment the nonfluorescent and mCherry-expressing populations. When BC187yb was co-cultured with YJM978ym, segmentation was applied to both mCherry and BFP signal to exclude debris and doublets. We optimized flow cytometry conditions to minimize the occurrence of doublets (<1%), and therefore segmentation with one or two fluorescent markers gave equivalent results. GAL1pr-YFP expression histograms were computed on the log_10_-transformed YFP signal of each segmented subpopulation.

Results of diauxic growth experiments (Figs. 2B, 2C, 5A, and 5B) are plotted so that time zero corresponds to when culture density was 10^6^ cells/ml rather than to inoculation time (S4B–S4D Fig.). This allows the glucose consumption rate of each strain to be compared by looking at the glucose exhaustion time (S11 Fig.). To determine the glucose exhaustion time for each dataset in Fig. 2, a line was fit to all glucose data points whose values lay between 0.01% and 0.05%, and the *x*-intercept of this line was taken as the time of glucose exhaustion. This method is more robust to measurement noise at low sugar concentrations than simply finding the time when concentration reaches some low threshold.

### Diauxic Growth Time-Course Measurements on Multiple Strains

To determine the timing of GAL pathway induction in multiple natural isolates (Fig. 3), we co-cultured GAL1pr-YFP-labeled versions of each “query” strain with a “reference” strain, YJM978ym, which contains a constitutive fluorescent protein, TDH3pr-mCherry, as well as a GAL1pr-YFP reporter (S1 Table; also see “Strains and Media”). Query strains were grown in liquid YPD for 16 h and then mixed with the reference strain YJM978ym at ratios of 1:4, 1:1, and 4:1 by volume. The mixed cultures were diluted 1:20 into YPD and grown for 4 h, and then diluted 1:200 in 2% raffinose and grown for 12 h. The raffinose cultures were then diluted 1:200 into 0.25% glucose + 0.25% galactose cultures split across 40 96-well plates. These were placed in a shaking incubator and allowed to grow for 8 h before beginning sampling. Every 15 min a plate was removed from the incubator, and its contents were mixed 1:1 with Tris-EDTA (pH 8.0) + 0.2% sodium azide to stop growth and protein synthesis, and incubated for 1 h at room temperature to allow fluorophore maturation. The 40 plates were then measured on the flow cytometer with the aid of a robotic arm.

We confirmed that the constitutive fluorophore does not affect the time of induction by co-culturing two YJM978 strains, with and without the TDH3pr-mCherry (S7B Fig.). We also compared the GAL reporter induction start time (*t*_low_) of BC187 and YJM978 when they were cultured separately and when co-cultured, and saw no significant difference for either strain (S7C Fig.). To check that growth rate differences between strains did not affect how quickly glucose was depleted, and therefore the timing of GAL reporter induction, we performed each co-culture experiment at three different initial ratios of query to reference strain, and obtained almost identical results (S7D and S7E Fig.). Therefore, this assay is robust to the presence and amount of reference strain, and we used the three inoculating ratios as replicates for data analysis.

To analyze population heterogeneity in GAL reporter induction (S7G–S7I Fig.), we computed the “ON fraction” as the fraction of cells with YFP signal greater than 1/32 of maximal median YFP. This threshold is just above the uninduced YFP level (S7G Fig.). The ON fraction increased monotonically in most of our strains. Some strains had a small pre-induced population at the start of sampling (S7H Fig.), consistent with the steady-state bimodality we have seen. Some strains did not seem to reach complete induction (ON fraction = 1), and in fact decreased in ON fraction because of an increasing YFP-off population toward the end of the time course (also see S7D Fig.). This was unlikely for biological reasons (all glucose and most galactose had been depleted at that point) and may reflect the presence of contaminants in the fixative. Our metrics were computed on data before this potential contaminant could reach appreciable concentrations, and therefore the potential contaminant does not affect the reported results. Likewise, before this point at least 90% of cells induced as one coherent population in all our strains (Fig. 7H and 7I), rather than as two subpopulations as seen by Venturelli et al. in strain W303 [23], which we did not assay here. The environmental and genetic determinants of induction time heterogeneity are potentially interesting to dissect in future experiments.

### Sudden Medium Shift Experiments

The medium shift experiment in Fig. 4A was performed by inoculating strains from a colony into liquid YPD, incubating for them 16 h, and then diluting them 1:500–1:8,000 into 2% glucose so that cell density was approximately 1 × 10^6^ after 12 h of further incubation. At this point, cultures were pelleted by centrifugation at 1,000*g* for 2 min and washed once in 2% galactose. The cultures were pelleted again and resuspended in 2% galactose, and a sample of cells was removed from each culture and measured on the flow cytometer every 20 min for 18 h. The same protocol was used when shifting strain YJM978ym from 0.125% glucose + 0.25% galactose to 0.125% glucose (S10 Fig.).

A similar experiment by New et al. using time-lapse microscopy after a glucose-to-maltose shift found that the average single-cell growth lag correlated with a metric similar to our diauxic lag time [17]. The apparent discrepancy between the findings of New et al. and our observations in Fig. 4A–4C is likely explained by differences in our metrics, the circuit studied (GAL versus MAL), and/or growth media. In particular, we used YNB, which contains no carbon sources other than glucose or galactose, whereas New et al. used YP, which contains peptone and yeast extract. We speculate that auxiliary carbon sources may modulate the response of cells to sudden primary carbon shifts, a potentially interesting effect for future investigation.

### Calculation of Induction Metrics

For both the diauxic growth (Figs. 2 and 3) and sudden medium shift experiments (Fig. 4A), we analyzed GAL1pr-YFP expression kinetics by calculating the time that a certain threshold value of median YFP signal was reached, and using these induction times to define other metrics (e.g., preparation time). These induction time calculations were always done by linear interpolation between two data points that bracketed the threshold YFP value. The threshold values of YFP signal were chosen to reflect the meaning of a given metric—for example, we considered the “start” of induction to be when YFP signal reached 2-fold above the basal expression of that strain (usually the initial value in a time course), and the “end” of induction to be when YFP signal was 4-fold below maximal expression. If the same metric was used in different experimental designs (e.g., execution time during diauxic growth or after medium shift), we occasionally chose different YFP thresholds to define the metric because of variation in the range of observed data. In general, however, our results were robust to the choice of threshold. For example, preparation time can be computed using a different definition of “mid-induction time” with almost identical results (S7F Fig.). For a detailed description of each metric used in this study, and when they can be compared across experiments, see S2 Table.

### Steady-State GAL Reporter Expression and Growth Rate Measurements

To measure the steady-state behavior of cells in defined glucose and galactose concentrations, we inoculated cells from a colony into liquid YPD for 16 h, diluted them in 2% raffinose and grew them for 20 h, and then inoculated them into glucose and/or galactose medium and grew them for at least 8 h before sampling. To maintain steady-state conditions, we diluted the cultures 1:3–1:10 with fresh medium every 2 h so that the culture density stayed below 10^6^ cells/ml (S12A Fig., light-colored lines). Based on the observed glucose consumption rates, this ensures that less than 10% of the glucose in a 0.0625% glucose medium is depleted. As a further check, we continued the experiment up to 48 h and found that GAL reporter expression reached steady-state at 8 h and stayed constant (S8 Fig.), indicating that our protocol was sufficient to prevent physiologically relevant changes in sugar concentrations.

To measure the steady-state relative growth rate and the absolute growth rate of strains BC187yb and YJM978ym (Figs. 5C and S12), we co-cultured them in various glucose and/or galactose media and sampled and diluted the cultures every 2 h for 12 h. We determined the growth rate difference (a.k.a. selection rate) by fitting a line to the log_2_ ratio of cell counts for each strain over time (Figs. 5C and S12B). We determined absolute growth rates from the same data by fitting a line to the log_2_ dilution-adjusted cell concentration (S12A and S12C Fig.; see also S4 Fig.). We obtained precise dilution factors by weighing culture tubes when empty and during each dilution. These experiments were done with *n* = 3–6 replicates. To compare growth rate differences at steady state to those from diauxic growth (Fig. 5A), we computed discrete derivatives of the log_2_ strain ratio at all consecutive data points in Phase I or Phase II, and compared their distribution with our steady-state measurements using a two-sample *t*-test (Fig. 5C).

### Single-Cell Measurements by Time-Lapse Microscopy

To prepare cells for time-lapse microscopy (S13 Fig.), we inoculated BC187ym cells from a colony into liquid YPD, grew them for 16 h, diluted them in 2% raffinose and grew them for 16 h, and then diluted them into pre-growth condition 0.125% glucose, 0.125% glucose + 0.25% galactose, or 0.25% galactose for 8 h to a density of 5 × 10^5^ cells/ml. Cells were then diluted 1:300 into 0.125% glucose + 0.25% galactose medium in wells (~1,000 cells/well) on a glass-bottom 96-well plate pre-coated with concanavalin A (Sigma) and left to settle for 1 h. BC187ym contained a GAL1pr-YFP promoter and a TDH3pr-mCherry marker for image segmentation. Imaging was performed on a Nikon Eclipse Ti inverted microscope through a 20× objective lens. Exposures were taken every hour for 4 h in bright field, YFP (ex. 500/24, em. 542/27), and mCherry (ex. 562/40, em. 641/75) channels, from 30 camera positions across two wells for each of the three pre-growth conditions, for a total of 90 camera positions across six wells. Image acquisition was controlled using custom MATLAB code using Micromanager/ImageJ.

Microscopy data were analyzed using custom MATLAB code. Microcolonies (clumps of 1–10 cells) were segmented in each mCherry image by applying a Gaussian blur to smooth cell boundaries, followed by a tophat filter to even out background, and thresholding to identify cells. Microcolonies were tracked across each time series by identifying overlapping areas. Colonies that split up, merged, entered, or exited the image during the acquisition time period were omitted from downstream analysis. Growth rate was computed as the change in log_2_ of a microccolony’s pixel area between the first and last time points, divided by elapsed time (4 h). YFP concentration was computed as the final average background-subtracted YFP signal per pixel area of a microcolony, where background YFP was taken as the median pixel intensity.

### Synthetic GAL Gene Induction Using the GEV System

Synthetic induction experiments (Fig. 6) were performed using three strains derived from FY5, a MATα S288C derivative (S1 Table) [52]. Strain SLYA32 (WT reference strain in Fig. 6C–6E) was transformed with a constitutive TDH3pr-mCherry-natMX4 (vector SLVA06) to allow flow cytometry segmentation. Strain SLYA39 (WT in Fig. 6B, query strain in Fig. 6E) was transformed with a GAL1pr-YFP-natMX4 reporter (vector SLVA64). Strain SLYH71 (GEV in Fig. 6) was transformed with a tandem GAL1pr-YFP-ACT1pr-GEV-hphNT1 replacing the HO locus (vector SLVD04). The GEV sequence was subcloned from vector pAGL, a generous gift from the Botstein lab [26]. To perform competitive growth experiments (Fig. 6C and 6D), query and reference strains were inoculated from single colonies into YPD, grown overnight, mixed 1:1 by volume, and then diluted 1:100 into YPD and grown 6 h to OD_600_ ~ 0.3. Then the cultures were concentrated 5× by centrifugation and diluted in triplicate 1:300 (1:60 dilution of cells) into 2% glucose or 2% glucose + 30 nM β-estradiol and grown 12 h to pre-induce. If needed (Fig. 6D), cells were shifted to 2% galactose by centrifugation at 3,000*g* for 2 min, washing in new medium, pelleting again, and resuspension. For the experiment in Fig. 6E, the above protocol was used, except query and reference strains were kept in separate cultures until the time of medium shift, and then mixed and resuspended together into new medium. The cultures were sampled immediately after the medium shift, and then every 30 min for 9 h, to measure the strain ratio by flow cytometry. The query strain in Fig. 6E (black line) was shifted from galactose medium back to the same medium, so the apparent delay in fitness advantage it exhibited may reflect a stress response to centrifugation and resuspension.

### Measuring Galactose Cost

To obtain the galactose cost (Figs. 7 and S14), we measured the growth rates of multiple strains in glucose and glucose + galactose. We co-cultured strains with the YJM978ym reference in 0.03125% glucose alone or 0.03125% glucose + 0.25% galactose (0.125% glucose in S14 Fig.), allowed them to grow for 8 h, and then measured the cell count ratio at two time points 4 h apart. To minimize glucose depletion, we inoculated cells so that their density at the end of the experiment did not exceed 3 × 10^6^ cells/ml. We computed the growth rate difference between the query and reference strains as

$$\Delta R=[\log_{2}(N_{\text{query,final}}∕N_{\text{ref,final}})-\log_{2}(N_{\text{query,initial}}∕N_{\text{ref,initial}})]∕4\;\text{h},$$

where *N* refers to the number of cells of a particular strain at a particular time point. We computed the absolute growth rate of the reference strain in each well as

$$R_{\text{ref}}=[\log_{2}(N_{\text{query,final}}∕N_{\text{ref,final}})-\log_{2}(N_{\text{query,initial}}∕N_{\text{ref,initial}})]∕4\;\text{h},$$

and then found the average and standard error of the mean (SEM) of reference strain growth rates across all wells of each condition as <*R*_ref,glu_> and <*R*_ref,glu+gal_> (see S2 Table). We computed the absolute growth rates of query strains as *R*_query_ = <*R*_ref_> + Δ*R* in each of the two conditions. Then we computed the galactose cost metric as (*R*_glu+gal_ − *R*_glu_)/*R*_glu_, where *R* denotes query the strain growth rate in each condition. Error bars are the SEM of galactose cost, computed from the SEM of measured Δ*R* and <*R*_ref_> values using standard uncertainty propagation formulas [55].

### Raw Data and MATLAB Code

The raw data and MATLAB analysis code used to generate all figures in this paper are deposited in the Dryad repository and are openly available via http://dx.doi.org/10.5061/dryad.39h5m [56].

## Supporting Information

S1 Fig Growth curves of all 43 strains assayed. Plots of log_2_(OD_600_) versus time for 43 strains, after subtracting background (0.03) from the raw OD_600_ readings. Two replicates are shown in each panel. Time axes have been adjusted so that OD_600_ = 2^−6^ at time zero, to exclude an initial interval of 0–12 h during which data can be noisy due to low OD_600_ (examples shown in S2B Fig.). The strains are shown sorted from shortest to longest diauxic lag time from top left to bottom right. Asterisks indicate strains shown in Fig. 1B and 1C based on their galactose growth rate (S3 Fig.). Plots outlined in green represent the 15-strain subset used for GAL reporter induction measurements (S1 Table; Figs. 3 and 4).
(EPS)

S2 Fig Diauxic lag and minimum mid-diauxic growth rate metrics correlate across replicates. (A) Measured optical density (i.e., absorbance at 600 nm) versus actual culture density, obtained by serial dilution of a yeast culture saturated under growth curve assay conditions. Dilution series were prepared in triplicate. OD_600_ was linear with culture density in this range, and displayed a background (*y*-intercept) value of ~0.03. (B) Example growth curves (top) and growth rate plots (bottom) for two strains. Light gray lines show raw discrete derivatives computed from the growth curve data (Materials and Methods), blue lines show cubic-spline fits to the discrete derivatives, and red lines show derivatives of the splines, or the smoothed second derivative of the growth curves. Both replicates are shown for each strain. Strain Bb32 (left) did not have a local growth rate minimum, and therefore its diauxic lag duration was defined to be zero, and its minimum mid-diauxic growth rate was defined to be the time of the inflection point in growth rate (Materials and Methods). More often, strains displayed a phenotype like that of SLYG78 (right), a S288C derivative, with a clear minimum rate during diauxic shift. (C) Scatter plots of the diauxic lag time (left) and minimum mid-diauxic growth rate (middle) across two replicate experiments, and between diauxic lag time and minimum rate (right). All three plots are strongly correlated, showing that our metrics were robust to measurement noise and that the continuous phenotypic variation in diauxic growth is not an artifact of the lag time metric used in Fig. 1. (D) Diauxic lag time and minimum mid-diauxic growth rate were calculated from a sliding-window average on the discrete derivatives of growth curves (as opposed to the cubic-spline fit used for Fig. 1). This method yielded almost identical results, showing that the metrics were not sensitive to the method of calculation.
(EPS)

S3 Fig Diauxic lag time is not correlated with growth rate in glucose-only or galactose-only medium. Scatterplots of diauxic lag time (top) and minimum mid-diauxic growth rate (bottom) versus steady-state growth rates in galactose alone (left) or in glucose alone (right). Steady-state growth rates were measured in a separate growth curve experiment (Materials and Methods). In general, the diauxic lag metrics correlated poorly with steady-state growth rates, suggesting that the phenotypic variation in diauxic growth cannot be solely explained by differences in glucose or galactose metabolism. Strains with growth rates between 0.5 and 0.6 doublings/hour in galactose (filled gray dots) are shown in Fig. 1B and 1C. These include BC187 (blue) and YJM978 (red).
(EPS)

S4 Fig Determination of absolute cell concentration by flow cytometric counting. (A) Absolute cell concentration as measured by flow cytometer versus actual relative culture density of a dilution series of a yeast culture, prepared in triplicate. Gray line shows predicted results extrapolated from the lowest density measurement, which agrees well with observed values. Absolute cell concentration was determined during the diauxic growth experiments in Figs. 2B, 2C, 5A, and 5B, and is plotted versus time for (B) BC187, (C) YJM978, and (D) a co-culture of BC187 and YJM978. Data for both replicates are shown—these are almost overlapping. The time axis is adjusted so that the culture is at 10^6^ cells/ml at time zero. This time was determined by interpolation on a linear fit to four consecutive data points. The same adjustment was applied to the time axis for all plots in Figs. 2 and 5.
(EPS)

S5 Fig Strain BC187 can consume galactose and glucose simultaneously. (A) Glucose and galactose concentrations versus time for a culture of BC187 (left) and a culture of YJM978 (right) from the same experiment as in Fig. 2. Both replicates are plotted. Time zero corresponds to a culture density of 10^6^ cells/ml. To determine whether either strain begins to consume galactose prior to glucose exhaustion, we computed the sugar depletion rate (B) by taking discrete derivatives (circles) of the sugar concentrations, or slopes between consecutive data points, for both replicate datasets. We then binned the time axis into 1-h intervals and computed, via a one-tailed *t*-test, the probability of observing the discrete derivatives in each time interval given a null hypothesis that the mean discrete derivative in that interval is zero or positive. Mean sugar depletion rate for each bin is shown as lines in (B), and the log_10_ *p*-value for the significance test is shown in (C). Dotted black lines in (C) indicate where *p* = 0.05. For BC187, there was a 2-h interval over which there was statistically significant depletion of both glucose and galactose, at a significance threshold of 0.05. By contrast, the time intervals of significant glucose and galactose depletion for YJM978 did not overlap temporally. These conclusions are robust to the width or position of time bins.
(EPS)

S6 Fig GAL1pr-YFP expression is highly variable across natural isolates in glucose + galactose but not in glucose alone. Steady-state GAL1pr-YFP expression histograms for 15 strains showing partial expression in 0.0625% glucose + 0.25% galactose (black), basal expression in 2% glucose (purple), and maximal expression in 2% galactose (orange). Additionally, the parent strains without a GAL1pr-YFP reporter cassette were assayed in 2% glucose (red). Partial expression varies widely across strains in glucose + galactose, yet YFP signal above autofluorescence is undetectable from most strains in glucose-only medium (compare purple and red histograms). A number of strains (YJM981, Y12-WashU, Y9-WashU, YJM975) display bimodal expression in glucose + galactose. Measurements were taken at steady state (as in Fig. 4D; see Materials and Methods); distributions are unsmoothed histograms of 20,000 or more cells.
(EPS)

S7 Fig Co-culture method to determine timing of GAL gene induction relative to glucose depletion. (A) Example scatterplot of YFP versus mCherry signal by flow cytometry. Reference strain and query strain cells are distinguished by mCherry (red versus gray), and the YFP of each subpopulation is used to compute induction time. (B) Median GAL1pr-YFP expression of YJM978 with or without constitutive fluorophore in a co-culture of the two strains. Both strains contain the GAL reporter, which is unaffected by the constitutive fluorophore. (C) Start time of GAL gene induction, *t*_low_, in BC187yb and YJM978ym cultured alone or in co-culture with each other, mean and range of two replicates. There is no significant difference in induction timing between separate and mixed cultures. (D) Median GAL1pr-YFP profiles for 15 strains from the co-culture experiment of Fig. 3. Query and reference strain were mixed at three initial ratios. Density plot in background shows full YFP distributions of the query strain for the 1:4 query:reference condition (except for strain SLYG78, where 4:1 is shown). (E) Scatterplot of preparation time from different inoculating ratios. Preparation time was nearly identical across different inoculating ratios. The three ratios were used as replicates in Fig. 3D and 3E. (F) Scatterplot of preparation time calculated as the time difference between query and reference strains at 1/32 or at 1/16 of maximal induction. The metric is robust to this difference (Spearman correlation = 0.97). (G) Definition of “ON fraction” as the fraction of cells with YFP signal higher than 1/32 of the maximal median YFP. (H) Possible ON fraction profiles. If a single population completely induces from basal to maximal (coherent induction), the ON fraction will increase monotonically from 0 to 1. If a culture splits into subpopulations with different induction times (early and late subpopulations), the ON fraction will increase in two distinct phases, as in Venturelli et al. [23]. If a subset of cells never induces (non-inducing subpopulation), the ON fraction will saturate below 1. (I) ON fraction versus time for the 15 strains in (D), from the 1:4 inoculation. Each strain is a different colored line, and strains BC187 and YJM978 are highlighted. Most profiles are consistent with coherent induction, although in some strains, a small subpopulation consisting of less than 10% of all cells may have pre-induction before sampling. In some strains, the ON fraction decreases after saturating (see also [D])—this is likely due to an experimental artifact (Materials and Methods).
(EPS)

S8 Fig GAL1pr-YFP expression reaches steady state after 8 h of growth in galactose medium. GAL1pr-YFP expression distributions over time in repressing (0.25% glucose), inducing (0.25% galactose), and mixed-sugar conditions for BC187yb (blue) and YJM978ym (red). Cultures were pre-grown in 2% raffinose to minimize the induction delay upon starting the experiment. Cells were diluted every 2 h to maintain a density of less than 10^5^ cells/ml. After 12 h, the dilution factor was increased, and dilution/sampling interval was increased to 12 h, and the cultures were monitored up to 48 h. In conditions where either strain induces, expression stops increasing after 8 h.
(EPS)

S9 Fig Strains induce GAL1pr-YFP at quasi-steady-state levels during gradual glucose depletion. (A) Scatterplot of median GAL1pr-YFP expression of query strains 3 h before reference strain mid-induction time (computed from data in Fig. 3) versus the median GAL1pr-YFP expression of the same strains growing at steady state in 0.0625% glucose + 0.25% galactose. (B) Steady-state GAL1pr-YFP distributions for strains BC187 and YJM978 (bottom) in glucose + galactose conditions chosen from different moments of diauxic growth (top schematic). BC187 induces at intermediate levels at steady state in glucose + galactose mixtures, rather than at basal or maximal levels, as would be expected if the level of GAL gene expression responds in a switch-like manner to decreasing glucose.
(EPS)

S10 Fig Pre-growth of YJM978 in a non-inducing galactose concentration accelerates GAL gene induction in subsequent medium shift. Median GAL1pr-YFP expression versus time for YJM978ym cells suddenly transferred from glucose to galactose (purple), or from glucose + galactose to galactose (black). This strain induces GAL genes significantly earlier (*p* = 0.008 by two-sample *t*-test) in response to sudden galactose induction when pre-grown in the presence of some galactose.
(EPS)

S11 Fig Short-lag strains reach saturation faster, but BC187 exhausts glucose more slowly than YJM978. (A) Example calculation of saturation time, which is defined as the time for a strain to grow from the diauxic shift to saturation. (B) Scatterplot of saturation time versus diauxic lag time. The two metrics are strongly correlated, showing that strains that have a shorter diauxic lag also reach saturation sooner after diauxic shift. (C) Time to exhaustion of glucose or galactose in cultures of BC187yb (blue) or YJM978ym (red). YJM978 exhausts glucose significantly before BC187, even though BC187 exhausts galactose—and therefore both sugars—much faster than YJM978 under the assay conditions. Data are mean and range of *n* = 2 replicates. *p*-Value was calculated by two-sample *t*-test.
(EPS)

S12 Fig Absolute and relative fitness of BC187 and YJM978. (A) Log2 absolute cell concentration versus time for strains BC187yb (blue) and YJM978ym (red) in 0.0625% glucose (left) and 0.0625% glucose + 0.25% galactose (right). Cultures were sampled every 2 h after they had reached steady-state GAL1pr-YFP expression. Cultures were periodically diluted so that raw cell densities (light color) did not exceed 2^20^ or 10^6^ cells/ml. Dilution-corrected data (dark color) were used to calculate growth rates. (B) Log2-ratio of BC187yb cell count to YJM978ym cell count in the same cultures as shown in (A). Relative fitnesses (i.e., growth rate differences) reported in Fig. 5C are computed from line fits to these plots. (C) Steady-state growth rates of BC187yb (blue) and YJM978ym (red) in 0.0625% glucose + 0.25% galactose, 0.0625% glucose, or 0.15% galactose, as determined by linear fit to plots as in (A). Bar graphs are mean and SEM of 3–6 replicates. *p*-Values are computed by two-sample *t*-test; “n.s.” indicates *p* > 0.05. (D) Steady-state GAL1pr-YFP expression distributions of BC187 (blue lines) and YJM978 (red lines) in the conditions from (C). Only one time point and replicate is shown; others had identical fluorescence distributions.
(EPS)

S13 Fig Single-cell growth rate correlates negatively with GAL1pr-YFP expression. (A) Example time-lapse microscopy images of BC187ym microcolonies (1–10 cells) at initial (top) and final (bottom) time points. Segmentation boundaries (red) were determined by analyzing mCherry images, and GAL1pr-YFP reporter concentration was determined as the final average YFP signal per pixel of each microcolony (Materials and Methods). (B) Scatterplots of growth rate versus YFP concentration for microcolonies pre-induced in 0.125% glucose (*n* = 165 microcolonies), 0.125% glucose + 0.25% galactose (*n* = 196), or 0.25% galactose (*n* = 223) prior to transfer to 0.125% glucose + 0.25% galactose for imaging. Growth rate displayed a significant negative correlation with GAL1pr-YFP concentration. (C) Distributions of growth rate (top) and YFP concentration (bottom) across microcolonies from the three pre-growth conditions. For clarity, plotted are smoothed probability densities estimated using a Gaussian kernel (Materials and Methods). *p*-Values are computed by a Kolmogorov-Smirnov test against the null hypothesis that growth rates of microcolonies from two pre-growth conditions have the same distribution.
(EPS)

S14 Fig Galactose cost and GAL gene expression change in a correlated way between different medium conditions. Scatterplot of galactose cost versus mean GAL1pr-YFP expression at steady state in two different sets of glucose or glucose + galactose media. Black circles are the same data as in Fig. 7B, whereas red circles are data obtained from 0.125% glucose and 0.125% glucose + 0.25% galactose, which induces GAL genes to a lesser extent. Gray arrows connect strains between the two conditions. Most arrows point toward the lower left, indicating that as galactose stays constant and glucose decreases (such as during diauxic growth), GAL gene expression increases at the same time that the growth cost due to the presence of galactose increases.
(EPS)

S1 Table Strains used in this study. This file contains three worksheets. Worksheet A lists the 43 natural isolates assayed by growth curves in Fig. 1. Worksheet B lists the GAL1pr-YFP reporter strains constructed from a subset of 15 natural isolates. Worksheet C lists strains used in time-lapse microscopy and synthetic GAL gene induction experiments.
(XLSX)

S2 Table Phenotypic measurements of natural isolates. This file contains four worksheets. Worksheet A summarizes the metrics used in the paper and describes how they are defined and interrelated. Worksheet B contains values of the diauxic lag time and minimum mid-diauxic growth rate metrics from both replicates of the growth curve experiments in Fig. 1. Worksheet C contains values of preparation time and other metrics measured on a subset of 15 natural isolates and used in Figs. 3 and 4. Worksheet D contains growth rate measurements used to determine the galactose cost, as well as GAL reporter expression data, plotted in Figs. 7 and S14.
(XLSX)

S1 Text All supporting figures and captions. Contains all the supporting figures and captions in one document.
(PDF)

## Abbreviations

GAL: galactose utilization
MAL: maltose utilization
SEM: standard error of the mean
WT: wild-type
YNB: yeast nitrogen base
