# Supplementary material for: Natural Variation in Preparation for Nutrient Depletion Reveals a Cost–Benefit Tradeoff
Source: PLoS Biol. 2015 Jan 27;13(1):e1002041. doi: 10.1371/journal.pbio.1002041 (PMC4308108; doi:10.1371/journal.pbio.1002041)
Supplement: S1 Text — Contains all the supporting figures and captions in one document. (PDF) [file pbio.1002041.s017.pdf]

Supporting Text 1: All Supporting Figures and Captions

This document contains Figures S1-S14 from Wang et al. 2014, “Natural Variation in Preparation for Nutrient Depletion Reveals a Cost-Benefit Tradeoff”.

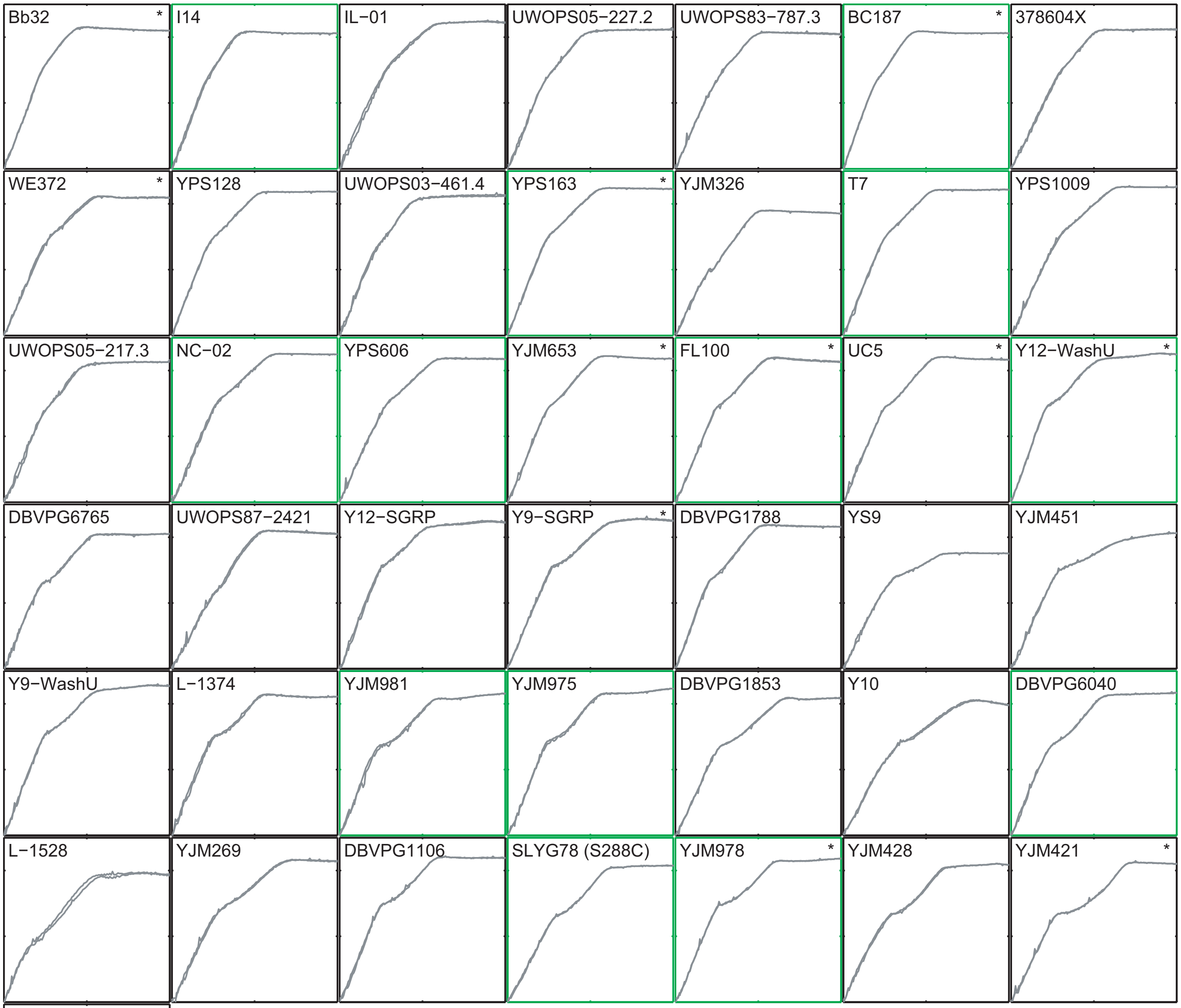

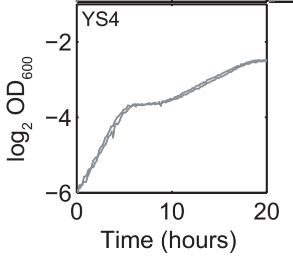

Figure S1. Growth curves of all 43 strains assayed. Plots of $\log_2$ (OD_{600}) versus time for 43 strains, after subtracting background (0.03) from the raw OD_{600} readings. Two replicates are shown in each panel. Time axes have been adjusted so that OD = 2^{-6} at time zero, to exclude an initial interval of 0-12 hours during which data can be noisy due to low OD (examples shown in Figure S2B). The strains are shown sorted from shortest to longest diauxic lag time from top left to bottom right. Asterisks indicate strains shown in Figure 1B-C based on their galactose growth rate (Figure S3). Plots outlined in green represent the 15-strain subset used for GAL reporter induction measurements (Table S1; Figure 3-4).

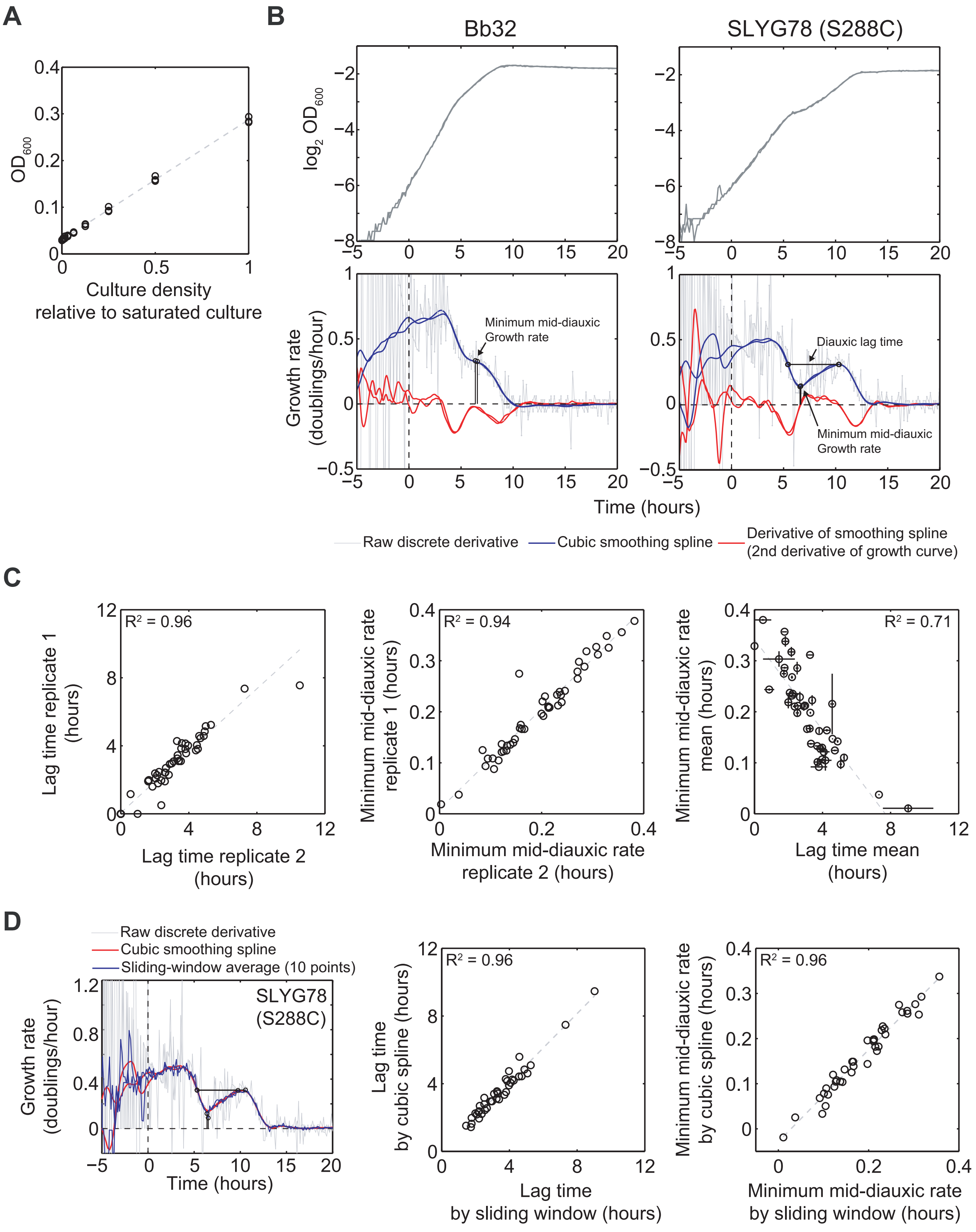

A
B
Bb32
SLYG78 (S288C)
OD600
Culture density
relative to saturated culture
log2 OD600
Growth rate
(doublings/hour)
Minimum mid-diauxic
Growth rate
Diauxic lag time
Minimum mid-diauxic
Growth rate
Time (hours)
Raw discrete derivative
Cubic smoothing spline
Derivative of smoothing spline
(2nd derivative of growth curve)
C
R2 = 0.96
Lag time replicate 1
(hours)
Lag time replicate 2
(hours)
R2 = 0.94
Minimum mid-diauxic rate
replicate 1 (hours)
Minimum mid-diauxic rate
replicate 2 (hours)
R2 = 0.71
Minimum mid-diauxic rate
mean (hours)
Lag time mean
(hours)
D
Raw discrete derivative
Cubic smoothing spline
Sliding-window average (10 points)
SLYG78
(S288C)
Growth rate
(doublings/hour)
Time (hours)
R2 = 0.96
Lag time
by cubic spline (hours)
Lag time
by sliding window (hours)
R2 = 0.96
Minimum mid-diauxic rate
by cubic spline (hours)
Minimum mid-diauxic rate
by sliding window (hours)

Figure S2. Diauxic lag and minimum mid-diauxic growth rate metrics correlate across replicates. (A) Measured optical density (i.e. absorbance at 600nm) versus actual culture density, obtained by serial dilution of a yeast culture saturated under growth curve assay conditions. Dilution series were prepared in triplicate. OD_{600} was linear with culture density in this range, and displayed a background (y-intercept) value of ~0.03. **(B)** Example growth curves (*top*) and growth rate plots (*bottom*) for 2 strains. Light gray lines show raw discrete derivatives computed from the growth curve data (Materials and Methods), blue lines show cubic spline fits to the discrete derivatives, and red lines show derivatives of the splines, or the smoothed 2nd derivative of the growth curves. Both replicates are shown for each strain. Strain Bb32 (*left*) did not have a local growth rate minimum, and therefore its diauxic lag duration was defined to be zero and its minimum mid-diauxic growth rate was defined to be the time of the inflection point in growth rate (Materials and Methods). More often, strains displayed a phenotype like that of SLYG78 (*right*), a S288C derivative, with a clear minimum rate during diauxic shift. **(C)** Scatter plots of the diauxic lag time (*left*) and minimum mid-diauxic growth rate (*middle*) across 2 replicate experiments, and between diauxic lag time and minimum rate (*right*). All 3 plots are strongly correlated, showing that our metrics were robust to measurement noise and that the continuous phenotypic variation in diauxic growth is not an artifact of the lag time metric used in Figure 1. **(D)** Diauxic lag time and minimum mid-diauxic growth rate were calculated from a sliding-window average on the discrete derivatives of growth curves (as opposed to the cubic spline fit used for Figure 1). This method yielded almost identical results, showing that the metrics were not sensitive to the method of calculation.

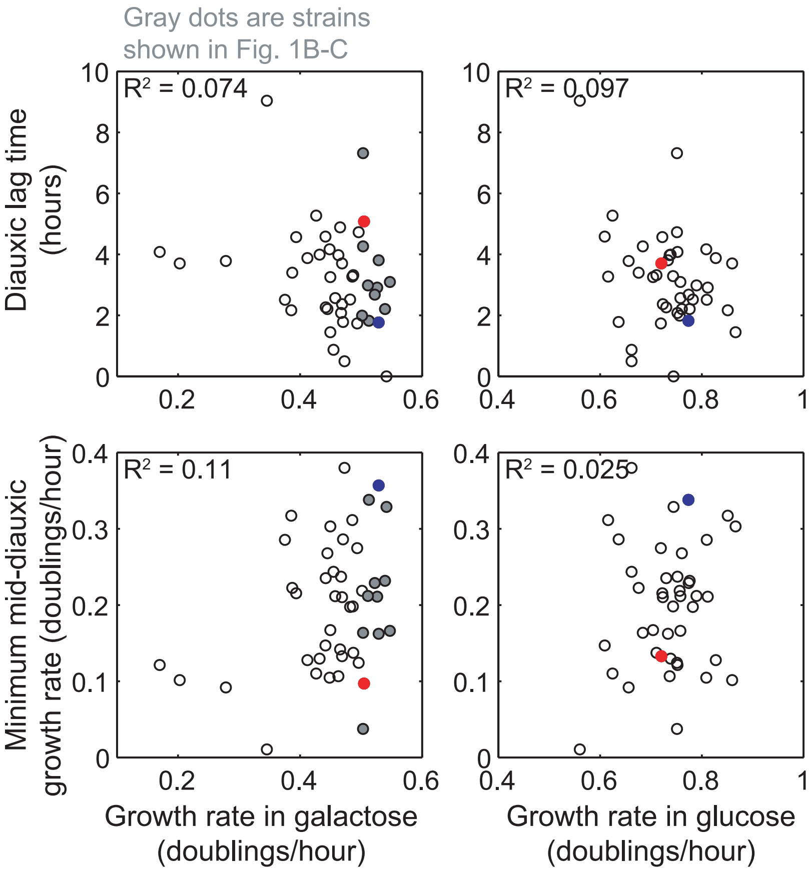

Figure S3. Diauxic lag time is not correlated with growth rate in glucose-only or galactose-only medium. Scatterplots of diauxic lag time (*top*) and minimum mid-diauxic growth rate (*bottom*) versus steady-state growth rates in galactose alone (*left*) or in glucose alone (*right*). Steady-state growth rates were measured in a separate growth curve experiment (Materials and Methods). In general, the diauxic lag metrics correlated poorly with steady-state growth rates, suggesting that the phenotypic variation in diauxic growth cannot be solely explained by differences in glucose or galactose metabolism. Strains with growth rates between 0.5 and 0.6 doublings/hour in galactose (filled gray dots) are shown in Figure 1B-C. This includes BC187 (blue) and YJM978 (red).

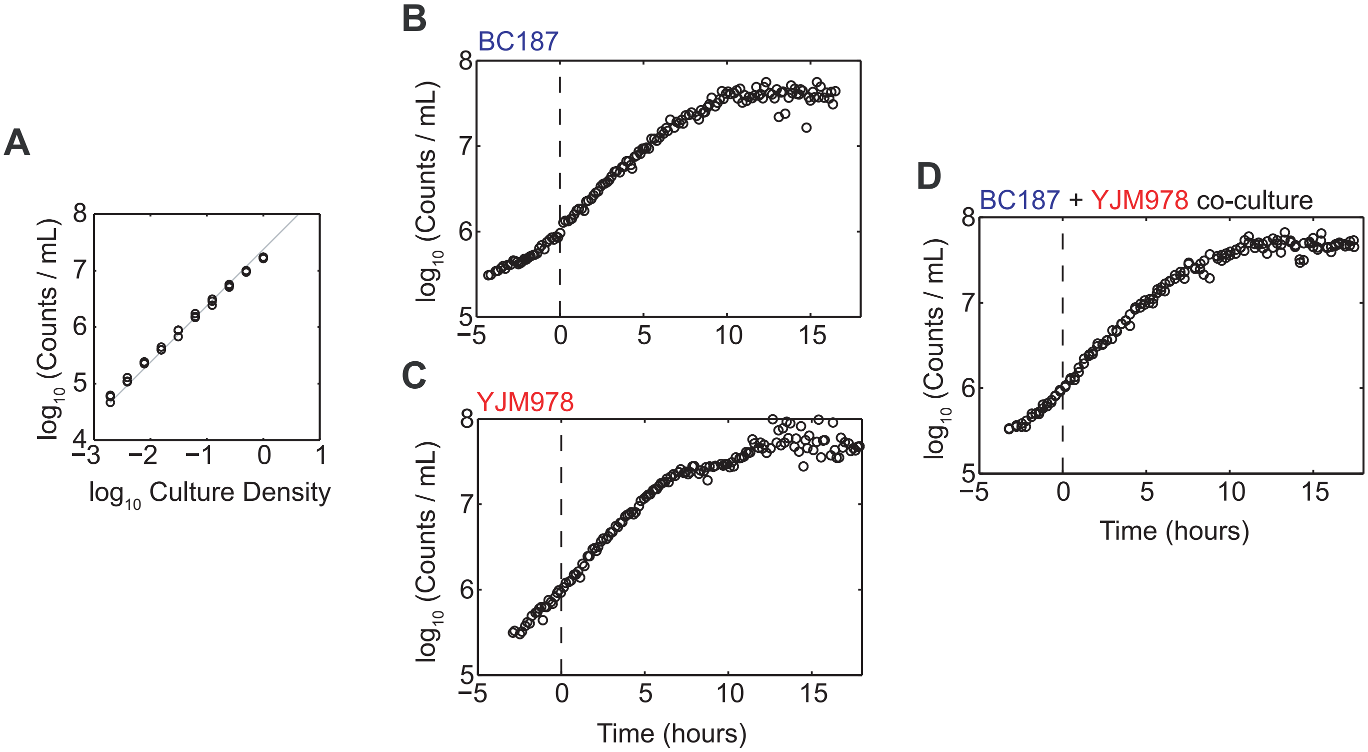

Figure S4. Determination of absolute cell concentration by flow cytometric counting. (A) Absolute cell concentration as measured by flow cytometer versus actual relative culture density of a dilution series of a yeast culture, prepared in triplicate. Gray line shows predicted results extrapolated from the lowest density measurement, which agrees well with observed values. Absolute cell concentration was determined during the diauxic growth experiments in Figure 2B-C and 5A-B, and is plotted versus time for **(B)** BC187, **(C)** YJM978, and **(D)** a co-culture of BC187 and YJM978. Data for both replicates are shown—these are almost overlapping. The time axis is adjusted so that the culture is at 10^6 cells/mL at time zero. This time was determined by interpolation on a linear fit to four consecutive datapoints. The same adjustment was applied to the time axis for all plots in Figures 2 and 5.

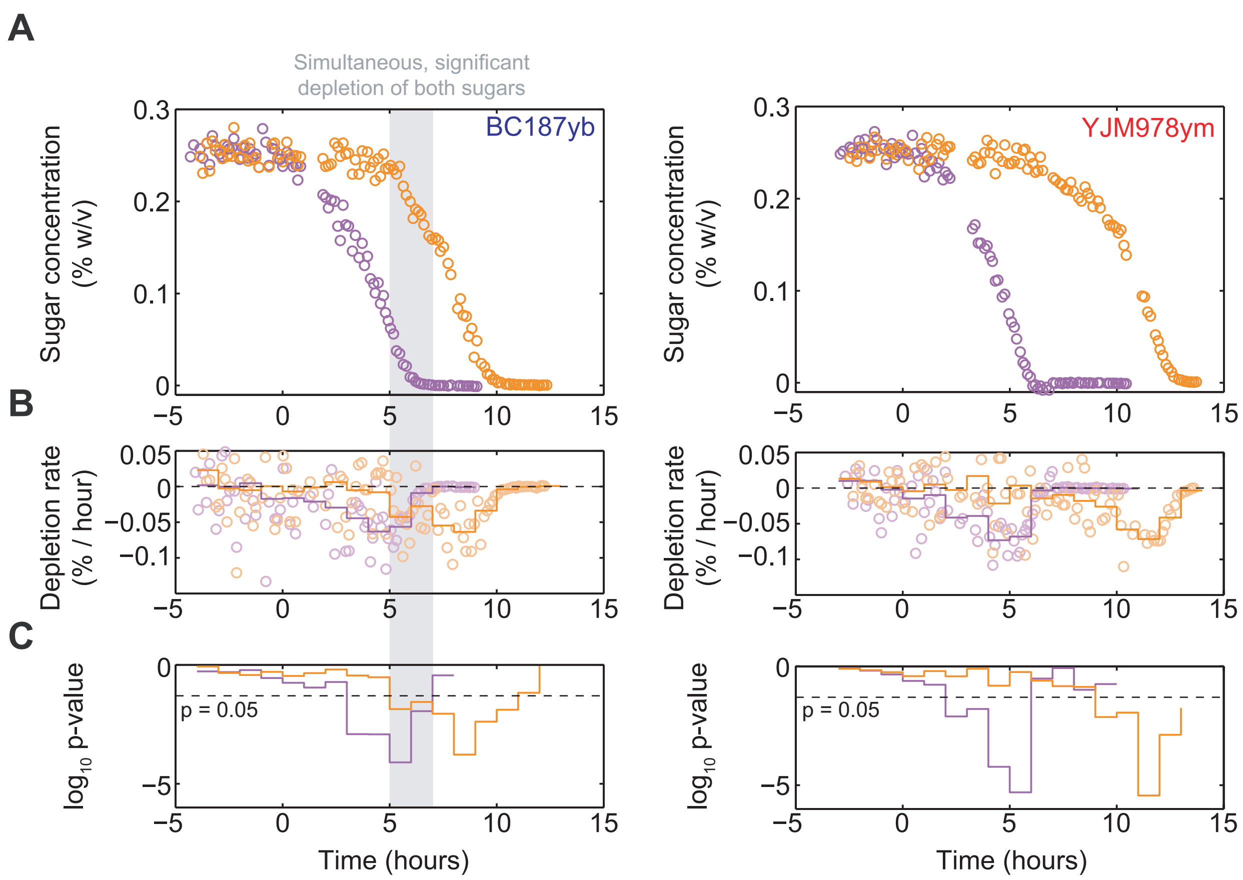

Figure S5. Strain BC187 can consume galactose and glucose simultaneously. **(A)** Glucose and galactose concentrations versus time for a culture of BC187 (*left*), and a culture of YJM978 (*right*), from the same experiment as in Figure 2. Both replicates are plotted. Time zero corresponds to culture density of 10^6 cells/mL. To determine whether either strain begins to consume galactose prior to glucose exhaustion, we computed the sugar depletion rate **(B)** by taking discrete derivatives (circles) of the sugar concentrations, or slopes between consecutive data points, for both replicate datasets. We then binned the time axis into 1-hour intervals and computed, via a one-tailed t-test, the probability of observing the discrete derivatives in each time interval given a null hypothesis that the mean discrete derivative in that interval is 0 or positive. Mean sugar depletion rate for each bin is shown as lines in (B) and the log10 p-value for the significance test is shown in **(C)**. Dotted black lines in (C) indicate where p=0.05. For BC187, there was a 2-hour interval over which there was statistically significant depletion of both glucose and galactose, at a significance threshold of 0.05. By contrast, the time intervals of significant glucose and galactose depletion for YJM978 did not overlap temporally. These conclusions are robust to the width or position of time bins.

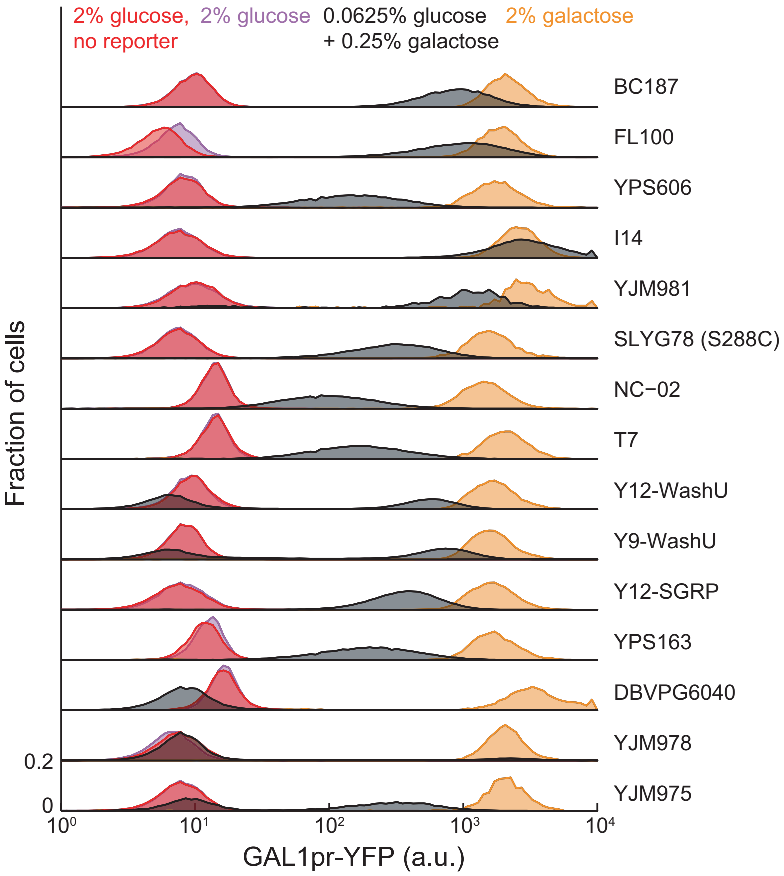

Figure S6. GAL1pr-YFP expression is highly variable across natural isolates in glucose + galactose but not in glucose alone. Steady-state GAL1pr-YFP expression histograms for 15 strains showing partial expression in 0.0625% glucose + 0.25% galactose (black), basal expression in 2% glucose (purple), and maximal expression in 2% galactose (orange). Additionally, the parent strains without a GAL1pr-YFP reporter cassette were assayed in 2% glucose (red). Partial expression varies widely across strains in glucose + galactose, yet YFP signal above autofluorescence is undetectable from most strains in glucose-only medium (compare purple and red histograms).
A number of strains (YJM981, Y12-WashU, Y9-WashU, YJM975) display bimodal expression in glucose+galactose. Measurements were taken at steady-state (as in Figure 4D; see Materials and Methods); distributions are unsmoothed histograms of 20,000 or more cells.

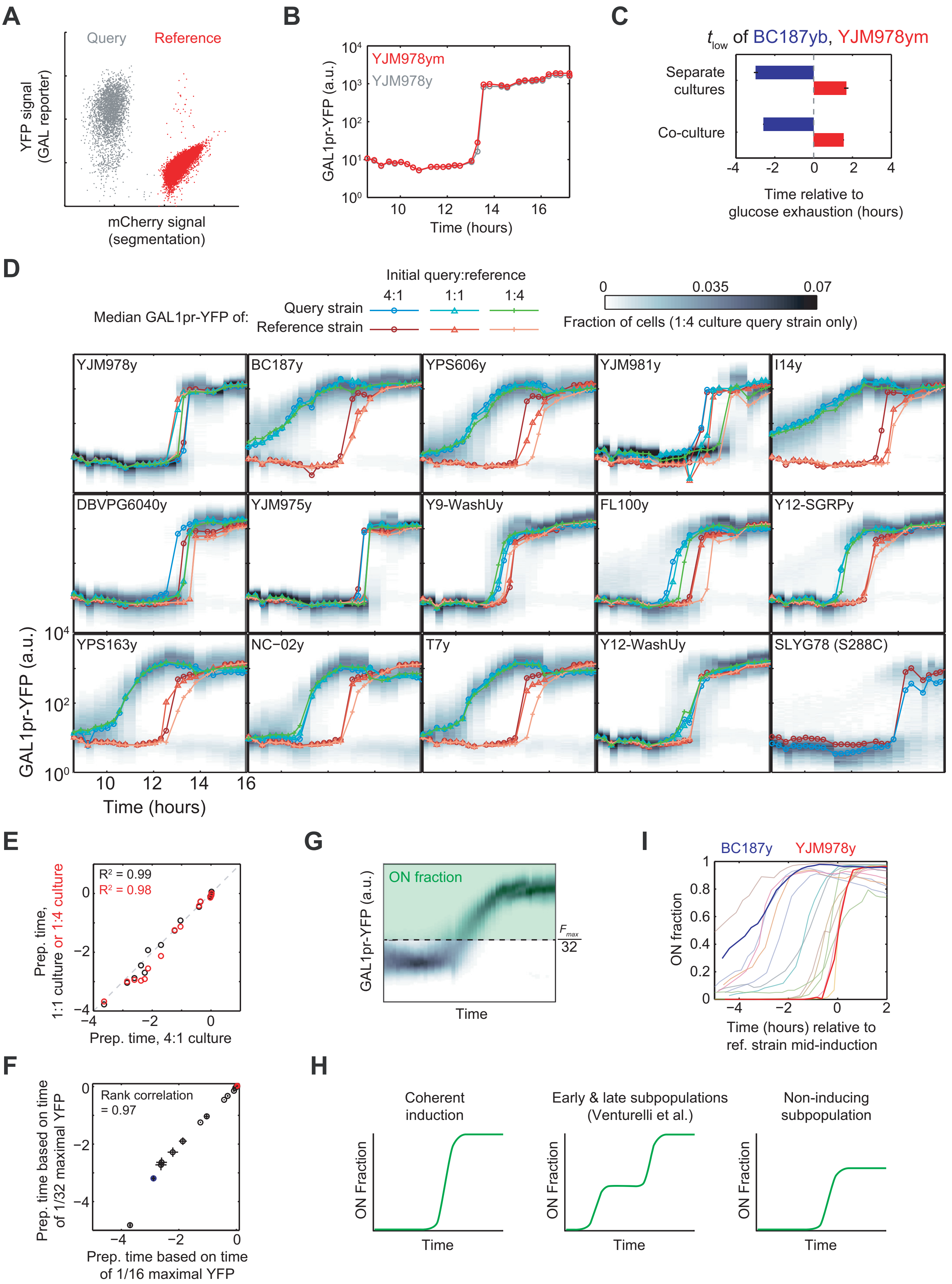
A
Query
Reference
YFP signal (GAL reporter)
mCherry signal (segmentation)
B
YJM978ym
YJM978y
GAL1pr-YFP (a.u.)
Time (hours)
C
t_{low} of BC187yb, YJM978ym
Separate cultures
Co-culture
Time relative to glucose exhaustion (hours)
D
Initial query:reference
4:1
1:1
1:4
Median GAL1pr-YFP of:
Query strain
Reference strain
0
0.035
0.07
Fraction of cells (1:4 culture query strain only)
YJM978y
BC187y
YPS606y
YJM981y
I14y
DBVPG6040y
YJM975y
Y9-WashUy
FL100y
Y12-SGRPy
YPS163y
NC−02y
T7y
Y12-WashUy
SLYG78 (S288C)
GAL1pr-YFP (a.u.)
Time (hours)
E
R^2 = 0.99
R^2 = 0.98
Prep. time, 1:1 culture or 1:4 culture
Prep. time, 4:1 culture
F
Rank correlation = 0.97
Prep. time based on time of 1/32 maximal YFP
Prep. time based on time of 1/16 maximal YFP
G
ON fraction
GAL1pr-YFP (a.u.)
F_{max} / 32
Time
H
Coherent induction
Early & late subpopulations (Venturelli et al.)
Non-inducing subpopulation
ON Fraction
Time
I
BC187y
YJM978y
ON fraction
Time (hours) relative to ref. strain mid-induction

Figure S7. Co-culture method to determine timing of GAL gene induction relative to glucose depletion. (A) Example scatterplot of YFP versus mCherry signal by flow cytometry. Reference strain and query strain cells are distinguished by mCherry (red vs gray) and the YFP of each subpopulation is used to compute induction time. **(B)** Median GAL1pr-YFP expression of YJM978 with or without constitutive fluorophore in a co-culture of the two strains. Both strains contain the GAL reporter, which is unaffected by the constitutive fluorophore. **(C)** Start time of GAL gene induction t_{low} in BC187yb and YJM978ym cultured alone or in co-culture with each other, mean and range of 2 replicates. There is no significant difference in induction timing between separate and mixed cultures. **(D)** Median GAL1pr-YFP profiles for 15 strains from the co-culture experiment of Figure 3. Query and reference strain were mixed at three initial ratios. Density plot in background shows full YFP distributions of the query strain for the 1:4 query:reference condition (except for strain SLYG78, where 4:1 is shown). **(E)** Scatterplot of preparation time from different inoculating ratios. Preparation time was nearly identical across different inoculating ratios. The three ratios were used as replicates in Figure 3D-E. **(F)** Scatterplot of preparation time calculated as the time difference between query and reference strains at 1/32 or at 1/16 of maximal induction. The metric is robust to this difference (Spearman correlation = 0.97). **(G)** Definition of "ON fraction" as the fraction of cells with YFP signal higher than 1/32 of the maximal median YFP. **(H)** Possible ON fraction profiles. If a single population completely induces from basal to maximal (coherent induction), the ON fraction will increase monotonically from 0 to 1. If a culture splits into subpopulations with different induction times (early & late subpopulations), the ON fraction will increase in two distinct phases, as in Venturelli et al. [23]. If a subset of cells never induce (non-inducing subpopulation), the ON fraction will saturate below 1. **(I)** ON fraction versus time for the 15 strains in (D), from the 1:4 inoculation. Each strain is a different colored line, and strains BC187 and YJM978 are highlighted. Most profiles are consistent with coherent induction, although in some strains, a small subpopulation consisting of less than 10% of all cells may have pre-induction before sampling. In some strains, the ON fraction decreases after saturating (see also panel D) – this is likely due to an experimental artifact (Materials and methods).

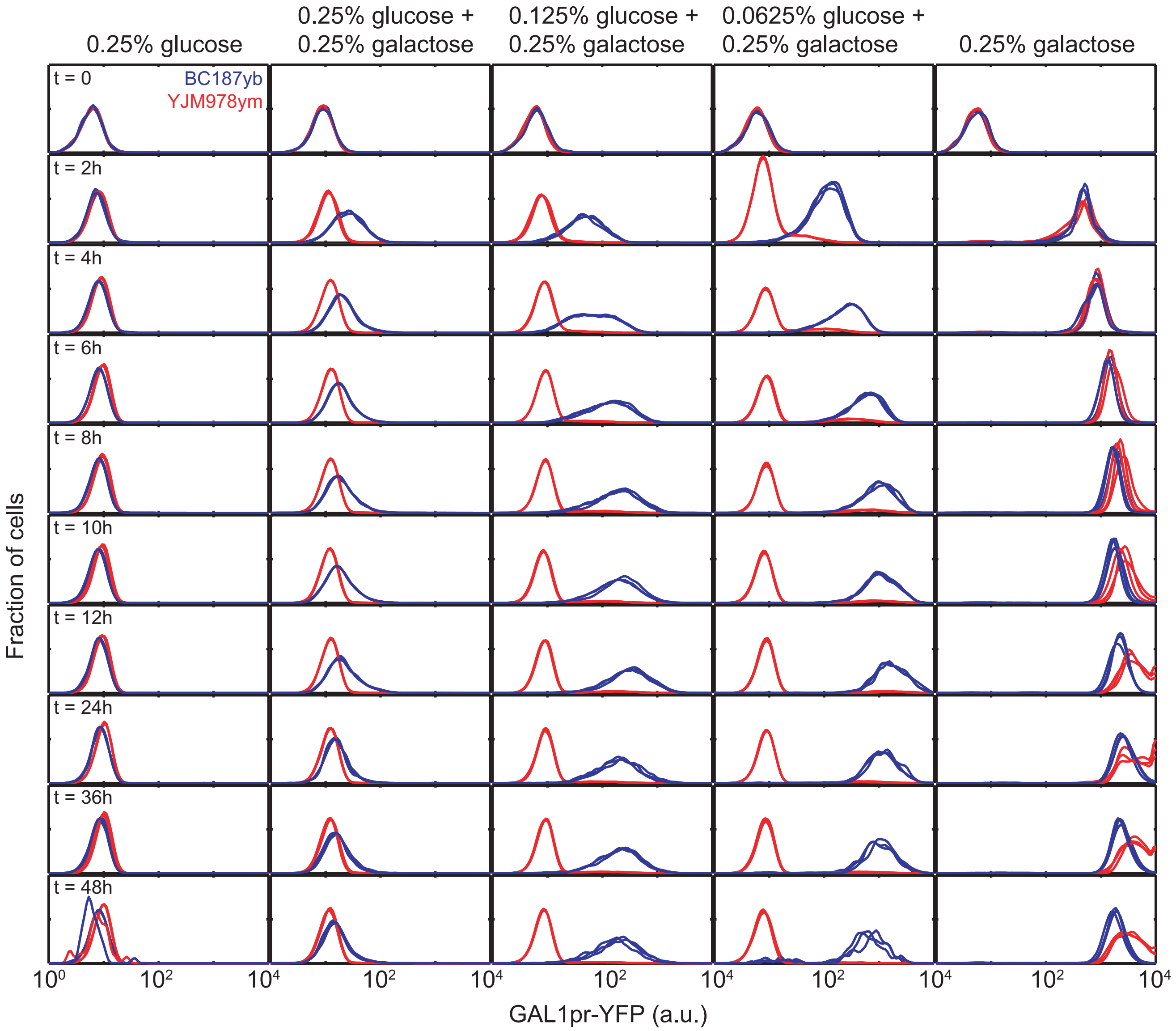

Figure S8. GAL1pr-YFP expression reaches steady-state after 8 hours of growth in galactose medium. GAL1pr-YFP expression distributions over time in repressing (0.25% glucose), inducing (0.25% galactose), and mixed-sugar (0.0625% glucose + 0.25% galactose) conditions for BC187yb (blue) and YJM978ym (red). Cultures were pre-grown in 2% raffinose to minimize the induction delay upon starting the experiment. Cells were diluted every two hours to maintain a density of less than 10^5 cells/mL. After 12 hours, the dilution factor was increased and dilution / sampling interval was increased to 12 hours, and the cultures were monitored up to 48 hours. In conditions where either strain induces, expression stops increasing after eight hours.

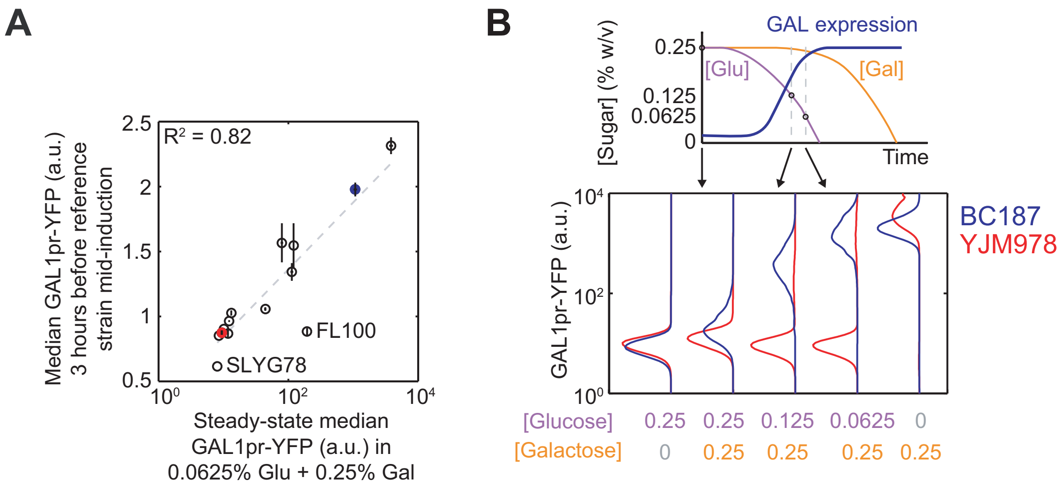

Figure S9. Strains induce GAL1pr-YFP at quasi-steady-state levels during gradual glucose depletion. (A) Scatterplot of median GAL1pr-YFP expression of query strains three hours before reference strain mid-induction time (computed from data in Figure 3) versus the median GAL1pr-YFP expression of the same strains growing at steady-state in 0.0625% glucose + 0.25% galactose. **(B)** Steady-state GAL1pr-YFP distributions for strains BC187 and YJM978 (*Bottom*) in glucose + galactose conditions chosen from different moments of diauxic growth (*Top schematic*). BC187 induces at intermediate levels at steady state in glucose + galactose mixtures, rather than at basal or maximal levels, as would be expected if the level of GAL expression responds in a switch-like manner to decreasing glucose.

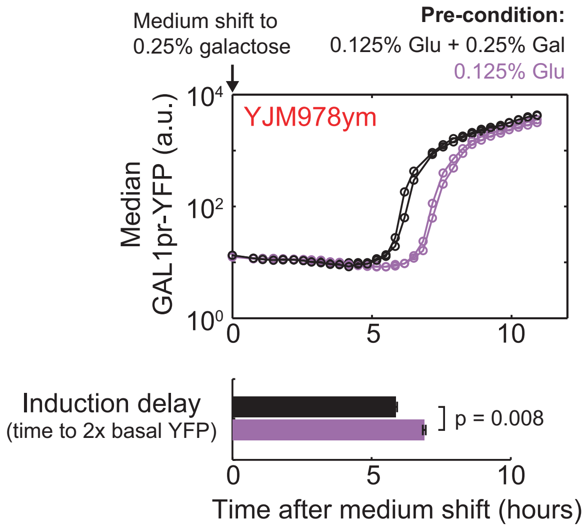

Figure S10. Pre-growth of YJM978 in a non-inducing galactose concentration accelerates GAL induction in subsequent medium shift. Median GAL1pr-YFP expression versus time for YJM978ym cells suddenly transferred from glucose to galactose (purple), or from glucose + galactose to galactose (black). This strain induces GAL genes significantly earlier (p=0.008 by 2-sample t-test) in response to sudden galactose induction when pre-grown in the presence of some galactose.

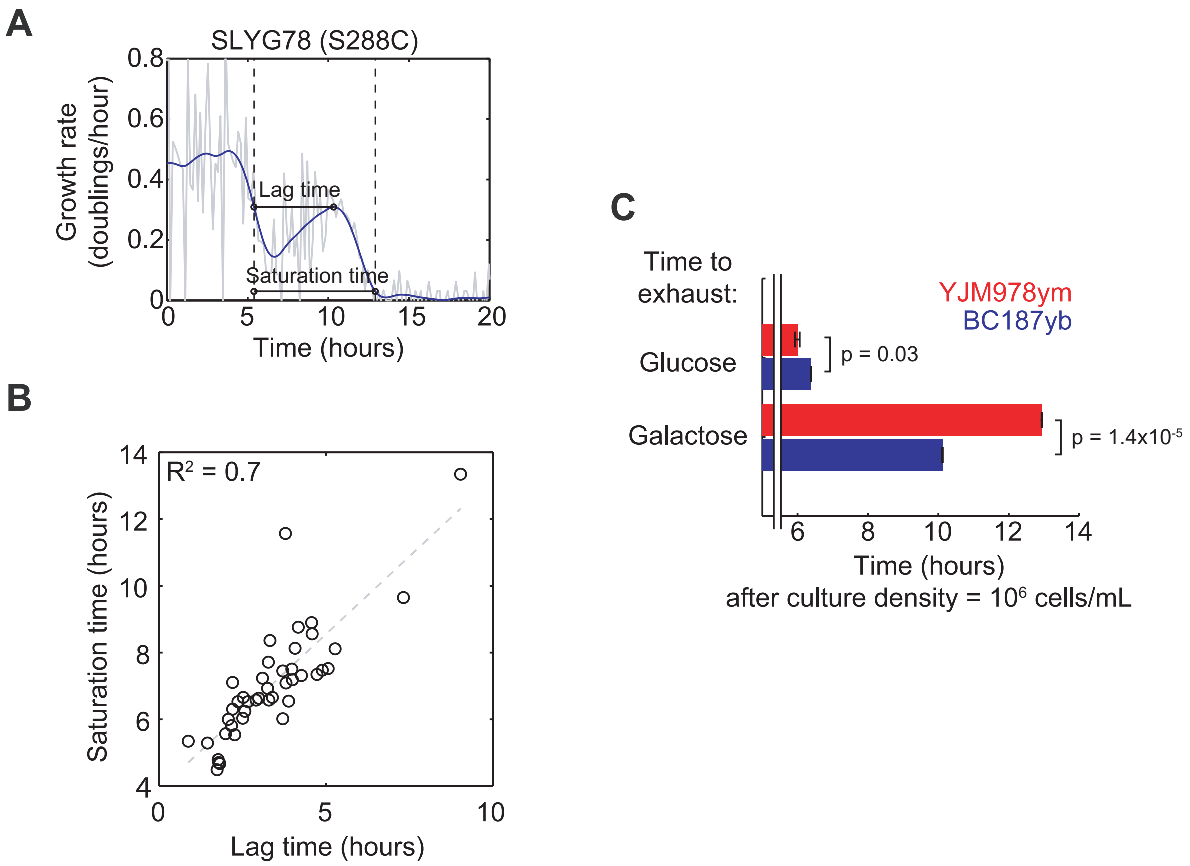

Figure S11. Short-lag strains reach saturation faster, but BC187 exhausts glucose more slowly than YJM978. (A) Example calculation of saturation time, which is defined as the time for a strain to grow from the diauxic shift to saturation. **(B)** Scatterplot of saturation time versus diauxic lag time. The two metrics are strongly correlated, showing that strains that have a shorter diauxic lag also reach saturation sooner after diauxic shift. **(C)** Time to exhaustion of glucose or galactose in cultures of BC187yb (blue) or YJM978ym (red). YJM978 exhausts glucose significantly before BC187, even though BC187 exhausts galactose—and therefore both sugars—much faster than YJM978 under the assay conditions. Data are mean and range of n=2 replicates. P-value was calculated by 2 sample t-test.

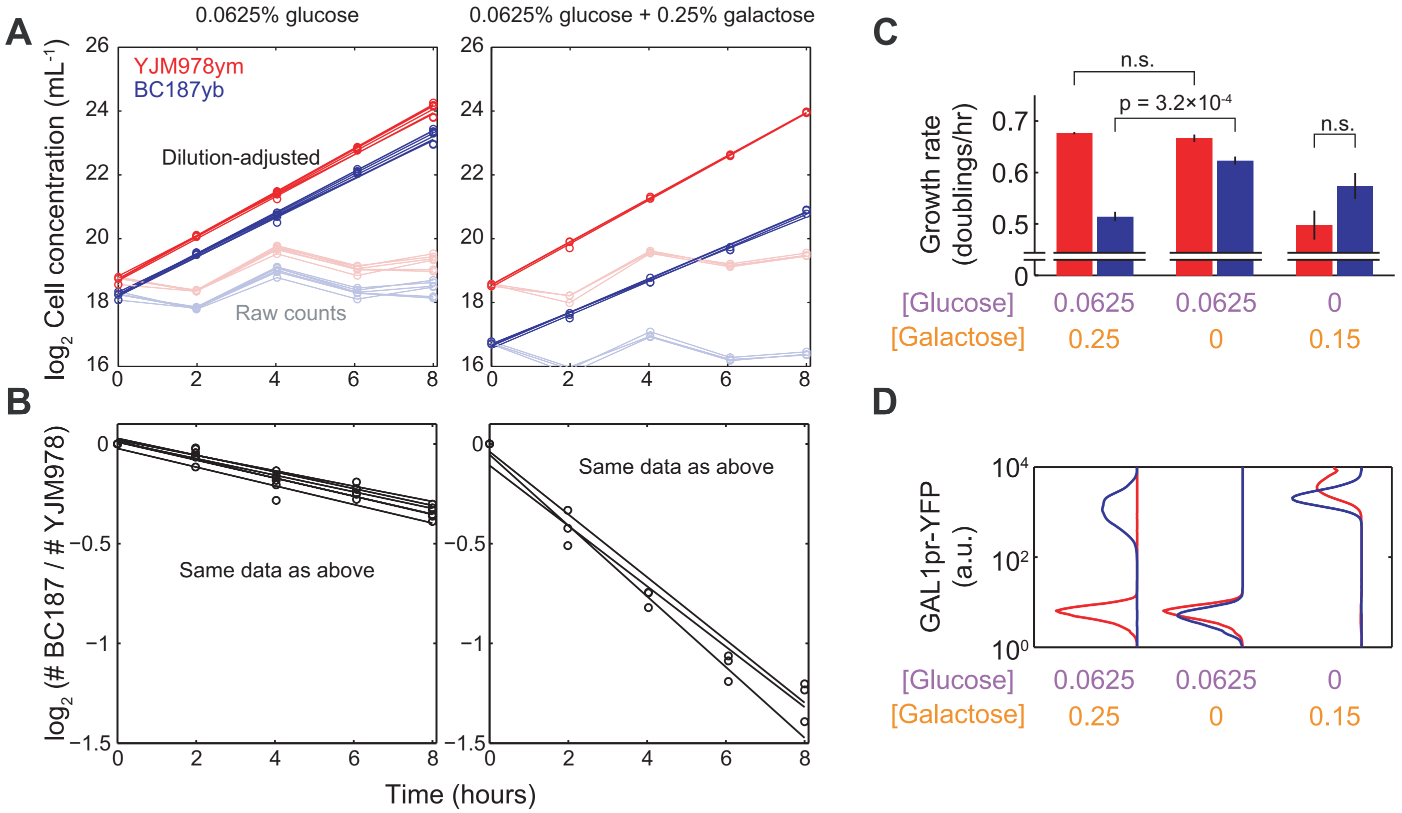

Figure S12. Absolute and relative fitness of BC187 and YJM978. (A) Log_2 absolute cell concentration versus time for strains BC187yb (blue) and YJM978ym (red) in 0.0625% glucose (left) and 0.0625% glucose + 0.25% galactose (right). Cultures were sampled every two hours after they had reached steady-state GAL1pr-YFP expression. Cultures were periodically diluted so that raw cell densities (light color) did not exceed 2^{20} or 10^6 cells/mL. Dilution-corrected data (dark color) were used to calculate growth rates. **(B)** Log_2-ratio of BC187yb cell count to YJM978ym cell count in the same cultures as shown in (A). Relative fitnesses (i.e. growth rate differences) reported in Figure 5C are computed from line fits to these plots. **(C)** Steady-state growth rates of BC187yb (blue) and YJM978ym (red) in 0.0625% glucose + 0.25% galactose, 0.0625% glucose, or 0.15% galactose, as determined by linear fit to plots as in (A). Bar graphs are mean and s.e.m. of 3-6 replicates. P-values are computed by 2-sample t-test; "n.s." indicates $p > 0.05$. **(D)** Steady-state GAL1pr-YFP expression distributions of BC187 (blue lines) and YJM978 (red lines) in the conditions from (C). Only one timepoint and replicate is shown; others had identical fluorescence distributions.

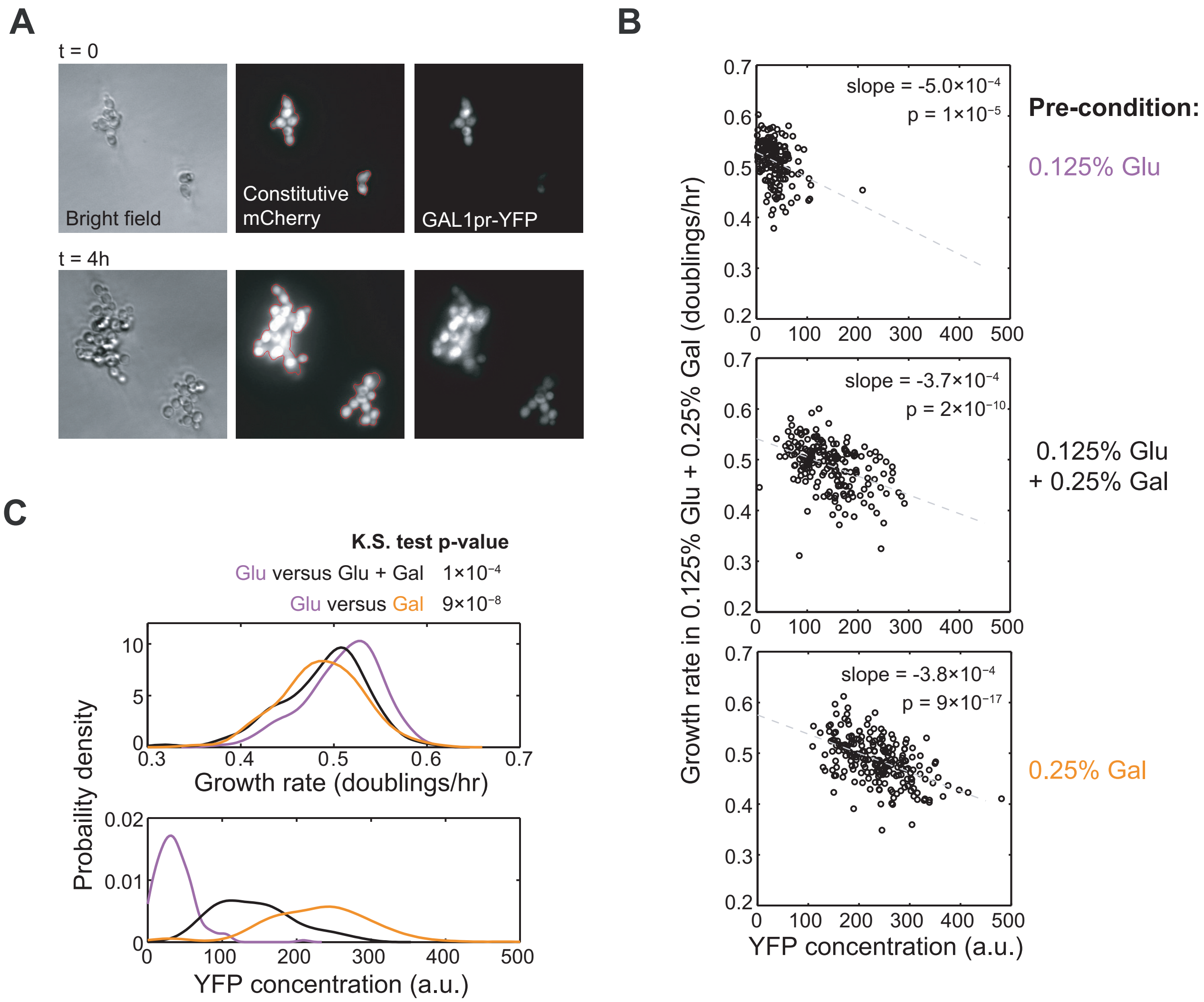

Figure S13. Single-cell growth rate correlates negatively with GAL1pr-YFP expression. (A) Example time-lapse microscopy images of BC187ym microcolonies (1-10 cells) at initial (top) and final (bottom) timepoints. Segmentation boundaries (red) were determined by analyzing mCherry images, and GAL1pr-YFP reporter concentration was determined as the final average YFP signal per pixel of each microcolony (Materials and methods). **(B)** Scatterplots of growth rate versus YFP concentration for microcolonies pre-induced in 0.125% glucose (n=165 microcolonies), 0.125% glucose + 0.25% galactose (n=196), or 0.25% galactose (n=223) prior to transfer to 0.125% glucose + 0.25% galactose for imaging. Growth rate displayed a significant negative correlation with GAL1pr-YFP concentration. **(C)** Distributions of growth rate (top) and YFP concentration (bottom) across microcolonies from the 3 pre-growth conditions. For clarity, plotted are smoothed probability densities estimated using a Gaussian kernel (Materials and methods). P-values are computed by a Kolmogorov-Smirnov test against the null hypothesis that growth rates of microcolonies from two pre-growth conditions have the same distribution.

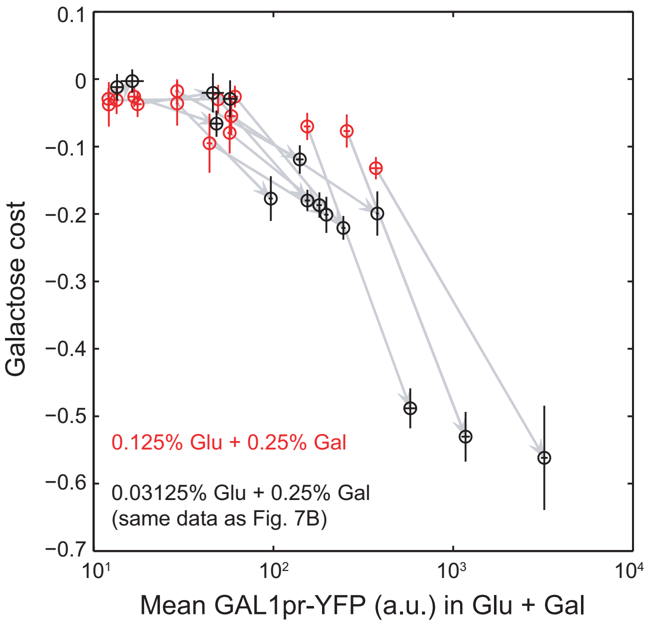

Figure S14. Galactose cost and GAL expression change in a correlated way between different media conditions. Scatterplot of galactose cost versus mean GAL1pr-YFP expression at steady-state in two different sets of glucose or glucose + galactose media. Black circles are the same data as in Figure 7B, whereas red circles are data obtained from 0.125% glucose and 0.125% glucose + 0.25% galactose, which induces GAL genes to a lesser extent. Gray arrows connect strains between the 2 conditions. Most arrows point toward the lower left, indicating that as galactose stays constant and glucose decreases (such as during diauxic growth), GAL expression increases at the same time that the growth cost due to the presence of galactose increases.
